# New cGMP analogues restrain proliferation and migration of melanoma cells

**DOI:** 10.18632/oncotarget.23685

**Published:** 2017-12-25

**Authors:** Eleonora Vighi, Andreas Rentsch, Philipp Henning, Antonella Comitato, Dorit Hoffmann, Daniela Bertinetti, Evelina Bertolotti, Frank Schwede, Friedrich W. Herberg, Hans-Gottfried Genieser, Valeria Marigo

**Affiliations:** ^1^ University of Modena and Reggio Emilia, Department of Life Sciences, 41125 Modena, Italy; ^2^ BIOLOG Life Science Institute Forschungslabor und Biochemica-Vertrieb GmbH, 28199 Bremen, Germany; ^3^ Department of Biochemistry, University of Kassel, 34132 Kassel, Germany

**Keywords:** PKG2, MNT1, SkMel28, cGMP, pVASP

## Abstract

Melanoma is one of the most aggressive cancers and displays high resistance to conventional chemotherapy underlining the need for new therapeutic strategies. The cGMP/PKG signaling pathway was detected in melanoma cells and shown to reduce migration, proliferation and to increase apoptosis in different cancer types. In this study, we evaluated the effects on cell viability, cell death, proliferation and migration of novel dimeric cGMP analogues in two melanoma cell lines (MNT1 and SkMel28). These new dimeric cGMP analogues, by activating PKG with limited effects on PKA, significantly reduced proliferation, migration and increased cell death. No decrease in cell viability was observed in non-tumor cells suggesting a tumor-specific effect. These effects observed in melanoma are possibly mediated by PKG2 activation based on the decreased toxic effects in tumor cell lines not expressing PKG2. Finally, PKG-associated phosphorylation of vasodilator-stimulated-phosphoprotein (VASP), linked to cell death, proliferation and migration was found increased and with a change of subcellular localization. Increased phosphorylation of RhoA induced by activation of PKG may also contribute to reduced migration ability of the SkMel28 melanoma cell line when treated with cGMP analogues. These findings suggest that the cGMP/PKG pathway can be envisaged as a therapeutic target of novel dimeric cGMP analogues for the treatment of melanoma.

## INTRODUCTION

Malignant melanoma is one of the most aggressive types of cancer and accounts for the majority of death related to skin cancer [1]. An early diagnosis allows surgical resection of melanoma but its tendency to metastasize causes melanoma progression to an invasive disease with high resistance to cytotoxic agents and poor prognosis in about 20% of patients [1]. The resistance to chemotherapy, radiotherapy and immunotherapy, which represents the main obstacle to a successful treatment, is possibly associated with low levels of spontaneous apoptosis compared to other tumor types [2]. New targeted therapies, such as BRAF inhibitors, contribute little to the overall patient survival [1]. A deeper characterization of the signaling pathways involved in melanoma initiation, progression and recurrence is thus highly needed to develop targeted therapies according to the genetic lesions that underlie each individual disease. On the basis of these principles, several new targeted agents are currently being developed and tested alone and in combination with conventional chemotherapies [1].

The cGMP/PKG signaling pathway has been linked to many cellular processes, including proliferation, differentiation and apoptosis in cells, as well as cancer cells [3]. cGMP has several intracellular targets: Protein kinase G (PKG) isozymes, cGMP-gated channels (CNGC) and it is hydrolyzed by specific phosphodiesterases (PDEs) [4]. PKG belongs to the family of serine-threonine kinases and is probably the most widely expressed cGMP effector protein [4]. Two genes were identified in mammals encoding PKG: *PRKG1* for PKG1α and PKG1β and *PRKG2* for PKG2 [5, 6]. PKG1α and PKG1β are widely expressed cytosolic enzymes that differ only in ∼100 amino acids in their amino-terminal sequences, whereas PKG2 is bound to the membranes and mainly expressed in the intestinal mucosa, in the breast tissue, in specific regions of the brain and in the retina [5].

The role of cGMP in cancer appears to be complex and dependent upon the type of tumor and the model system investigated [3]. Both pro- and anti-cancer effects of cGMP have been reported. For example, the activation of the cGMP/PKG pathway can induce apoptosis in colon cancer cells [7], breast cancer cells [8–11], pancreatic adenocarcinoma cells [12], gastric cancer cells [13] and head and neck squamous carcinoma cells [14]. Specific activation of PKG1 in melanoma was shown to trigger MAPK signaling and promote melanoma growth *in vitro* and *in vivo* [15]. Several components of the cGMP/PKG pathway, such as PDE6 and CNGC, are expressed by melanoma cells, nonetheless few studies are available on the cGMP signaling pathway in melanoma [16, 17]. Activation of PKG1α and/or PKG1β has been linked to melanoma progression and aggressiveness [15, 18–21] but, to our knowledge, the role of PKG2 has not been characterized yet. Interestingly, anti-tumor properties have been associated with PKG2 activation in breast cancer [8], gastric cancer [13] and glioma [22]. PKG2 expression was found downregulated in breast tumors compared to normal tissue, supporting the antitumor activity of this kinase [8].

In this study, we assessed the expression of the different PKG isoforms in two melanoma cell lines with the aim of testing the effects of activators of the cGMP/PKG pathway in these cells. All 3 PKG isoforms were found expressed in both melanoma cell types but at different levels. We exposed the cells to 6 different cGMP analogues to activate PKG and assessed cell viability and mobility. We identified 2 compounds reducing melanoma cell viability and mobility and found that they differently affect the phosphorylation pattern of the vasodilator-stimulated phosphoprotein (VASP), a cytoskeletal protein linked to apoptosis, proliferation and migration.

## RESULTS

### Expression of PKG isoforms in MNT1 and SkMel28 cells

In this study, we analyzed two human melanoma cell lines: MNT1 derived from pigmented pediatric melanoma and SkMel28 derived from white adult melanoma and we characterized them on the BRAF V600E variant, the most common mutation in melanoma. MNT1 cells bear the BRAF V600E mutation in heterozygosis (T>A, Supplementary Figure 1A), whereas SkMel28 cells carry the BRAF V600E mutation in homozygosis (Supplementary Figure 1B), as reported in the ATCC specification.

**Figure 1:**
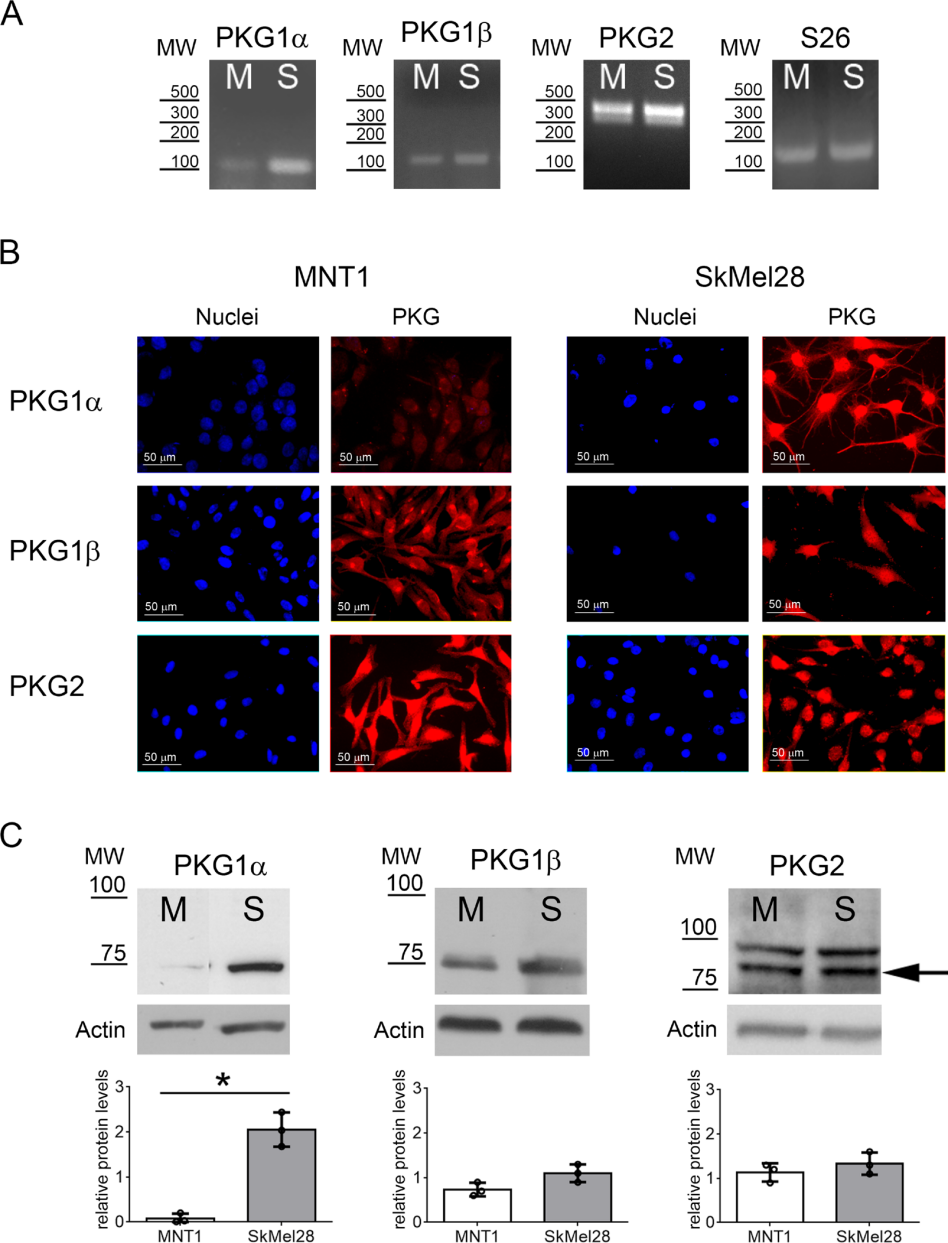
PKG expression in melanoma cell lines (**A**) Expression of PKG1α, PKG1β and PKG2 in MNT1 (M) and SkMel28 (S) was assessed by RT-PCR. *S26* was analyzed as reference gene. Primers for PKG2 could detect the two major isoforms that are expressed in both melanoma cell lines. (**B**) Immunofluorescence analysis using specific antibodies for the three PKG isozymes. (**C**) Immunoblotting using specific antibodies for the three PKG isozymes in MNT1 (M) and SkMel28 (S). Quantifications of protein levels detected by immunoblotting are shown below of each blot. The arrow indicates the band at the expected molecular weight for PKG2 (86 kDa). MW= Molecular weight: in RT-PCR as base pairs (bp) (A) and in immunoblotting as kDa (C).

We then evaluated the expression of the different PKG isoforms at mRNA and protein levels. All three PKG isoforms were present in MNT1 and SkMel28 cells (Figure 1A–1C). We could also detect the two major variants of PKG2, variant 1 and variant 6, in both cell lines (Figure 1A). Quantitative protein analysis by immunoblotting showed that PKG2 and PKG1β are expressed at similar levels in the two melanoma cell lines (*p* > 0.05), whereas expression of PKG1α is higher in SkMel28 than in MNT1 (*p* = 0.028) (Figure 1C). Similar subcellular distribution in the two cell lines was observed for PKG1α and PKG1β (Figure 1B), but confocal analysis showed that PKG2 is associated to different intracellular membranes in the two cell lines: in MNT1, it was associated to endoplasmic reticulum (ER) but not to mitochondrial membranes (Supplementary Figure 2A–2B), whereas in SkMel28 PKG2 was bound to both mitochondrial and ER membranes (Supplementary Figure 2C–2D).

As controls we analyzed a human non-tumor cell line derived from skin keratinocytes, i.e. HaCaT, expressing all PKG isoforms as shown by immunoblotting (Supplementary Figure 3A), and two tumor cell lines with different PKG expression patterns. SHSY5Y cells, derived from human neuroblastoma, expressed PKG1α and PKG1β but not PKG2 and A673 cells, derived from human Ewing's sarcoma, expressed only PKG1α (Supplementary Figure 3A).

### Synthesis of PKG activators

**Figure 2:**
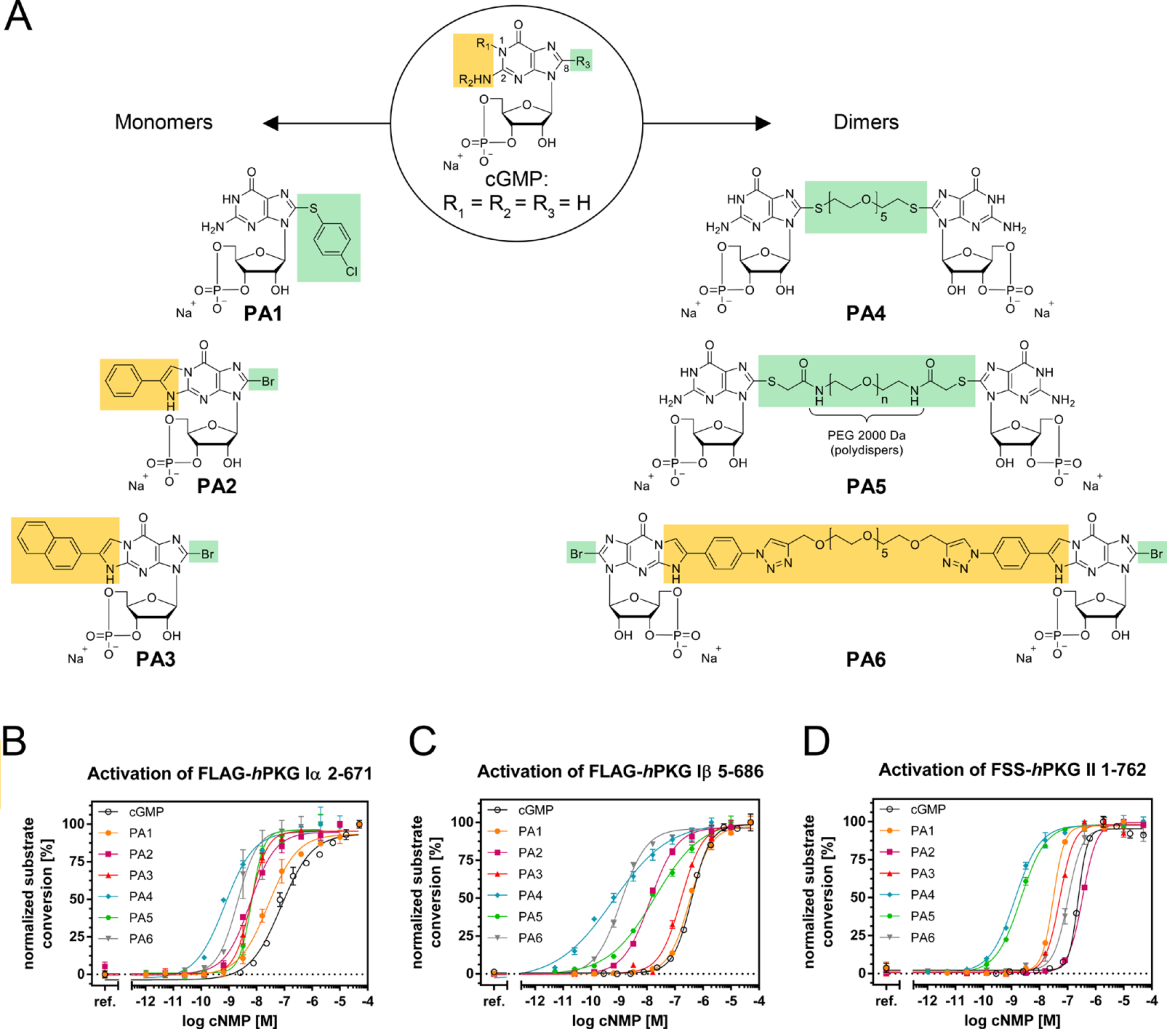
Structures of monomeric and dimeric cGMP analogues displayed in the sodium salt form (**A**) R1+R2 resemble the β-phenyl-1,N^2^-etheno (PET) modification, while R3 refers to the 8-position (C8) of cGMP and contains either bromine as residue or the linking moiety (including spacer and coupling/bridging functions). (**B–D**) Normalized activation curves of (B) PKG1α, (C) PKG1β and (D) PKG2 with cGMP and PA1-PA6.

To reveal whether PKG can be a target to restrain cell viability in melanoma, we tested cGMP analogues, which are known PKG activators and commercially available, as well as newly synthesized compounds (Figure 2A; Supplementary Table 1).

Our synthetic approach focused on the so far rarely studied class of polymer linked dimeric (PLD) cGMP analogues. For a small homologous set of such PLDs, modified only in terms of their spacer length in between the two cGMP units, Kramer and Karpen [23] reported strong PKG1α and CNG channel activation potential and suggested the spacer length to be a crucial modifier. In an attempt to expand the PLD idea to further targets of the cGMP signaling cascade, including PKG1β and PKG2, we produced three new PLDs (PA4, PA5 and PA6, see Figure 2A). For PA4 and PA5 we followed a similar strategy as previously reported [23], wherein we introduced PEG spacers of different lengths via a thio-function at the C8 position. We chose a spacer with 5 ethylene oxide units for PA4 and 45 ethylene oxide units (average number referring to a polydispers spacer with average MW of 2000 Da) for PA5. In order to circumvent a possible connection via the undesired N7 position by using vinyl sulfonyl substituted spacer reagents [24], the original synthetic protocol [23] was replaced by new more regioselective methods.

Nucleobase derivatization of PLDs has not been performed before, but presents a promising motive for optimizing the activation potential. In PA6, we therefore included a common modifier of the nucleobase, the β-phenyl-1,N^2^-etheno (PET) group, in both nucleobases (the PET group is also present in monomeric PA2 and as a derivative in PA3). Furthermore, in this derivative, we established a new linkage of the cGMP units via said PET groups, using a spacer with similar length as in PA4.

The six PA cGMP analogues had distinctive activation constants in the range of < 1 nM up to 320 nM on the respective PKG1α, PKG1β and PKG2 isozymes, as tested *in vitro* on purified proteins (Table 1 and Figure 2B–2D) [25].

**Table 1:** PKG1α, PKG1β and PKG2 activation constants by PA1, PA2, PA3, PA4, PA5 and PA6

	**FLAG-*h*PKG1α (n ≥ 3)**						
	**cGMP**	**PA1**	**PA2**	**PA3**	**PA4**	**PA5**	**PA6**
mean ± SD	49 ± 4 nM	26 ± 5 nM	4.6 ± 0.2 nM	5.7 ± 1.0 nM	< 0.8 nM	7.7 ± 1.3 nM	< 2 nM
	**FLAG-*h*PKG1β (n ≥ 3)**						
	**cGMP**	**PA1**	**PA2**	**PA3**	**PA4**	**PA5**	**PA6**
mean ± SD	430 ± 30 nM	320 ± 20 nM	16 ± 3 nM	13 ± 4 nM	< 1 nM	21 ± 2 nM	< 1 nM
	**FSS-*h*PKG2 (n ≥ 3)**						
	**cGMP**	**PA1**	**PA2**	**PA3**	**PA4**	**PA5**	**PA6**
mean ± SD	250 ± 20 nM	28 ± 3 nM	300 ± 45 nM	58 ± 5 nM	< 1 nM	2.1 ± 0.6 nM	110 ± 30 nM

### Effect of PKG activators on cell viability

**Figure 3:**
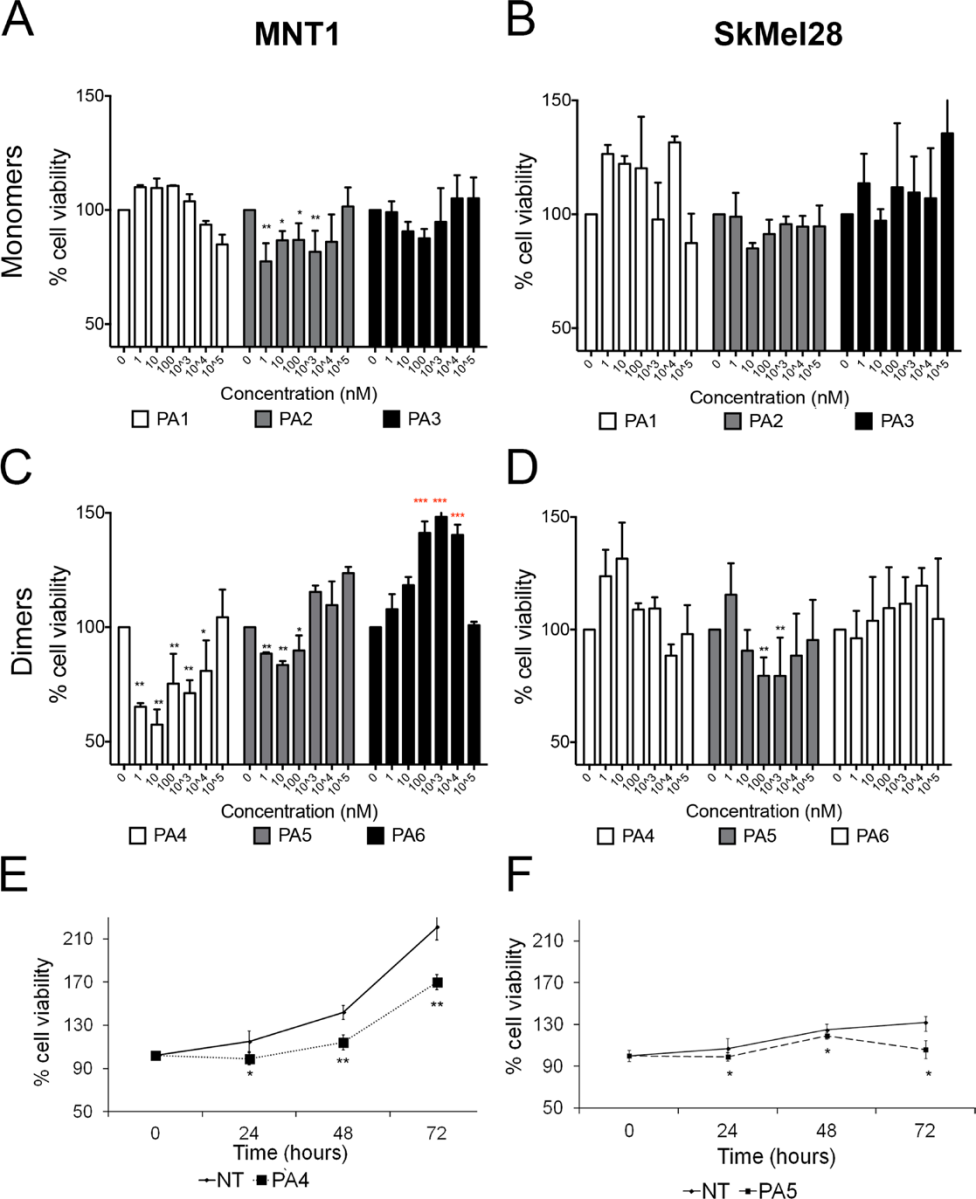
Cell viability assay after treatment with cGMP analogues In all experiments values of not treated cells (NT) were set as 100% cell viability. (**A–B**) Dose responses in MNT1 (A) and in SkMel28 (B) treated with monomeric cGMP analogues for 24 h. (**C–D**) Dose-responses in MNT1 (C) and in SkMel28 (D) treated with dimeric cGMP analogues for 24 h. (**E**) Time-course assay in MNT1 cells treated with PA4 at 1 μM (^*^p = 0.04). (**F**) Time-course assay on SkMel28 treated with PA5 at 1 μM (*p* = 0.03^*^). Error bars: SD. Statistical comparison: in A-B-C-D NT vs treated at each concentration with Student's unpaired two-tailed *t*-test; in E-F NT vs treated using Student's paired two tailed *t*-test. Significance levels: *p* < 0.05 = ^*^p < 0.01 = ^**^p < 0.001 = ^***^. Red asterisks indicate significant increase of cell viability.

To evaluate the effects of PKG activation on melanoma cell viability we tested three monomeric cGMP analogues that acted as PKG activators, i.e. PA1 (8-pCPT-cGMP) [9], PA2 (8-Br-PET-cGMP) [9] and PA3 (8-Br-(2-N)-ET-cGMP) [26]. PA1 and PA2 are commercially available and well-accepted tools in the cGMP-signaling field. In MNT1 cells only PA2 was able to reduce cell viability by around 25% after 24 h treatment (Figure 3A). None of the monomeric cGMP analogues had a significant effect on SkMel28 cell viability at the tested concentrations after 24 h of treatment (Figure 3B).

We then treated these cells for 24 h with three new dimeric cGMP analogues (PA4 – PA6) and found that PA4 and PA5 reduced viability of MNT1 (Figure 3C). PA4 was identified as the most potent compound and was thus selected for the following experiments. Only PA5, in a concentration range between 0.1–1 μM, reduced cell viability of SkMel28 cells (Figure 3D). PA6 had no effect on SkMel28 cells viability but significantly increased viability of MNT1 cells.

None of the tested cGMP analogues decreased cell viability of non-tumor cells HaCaT (Supplementary Figure 3B). On the contrary PA4 and PA5 significantly increased cell viability of SHSY5Y in a range from 10 nM to 10 μM (Supplementary Figure 3C) whereas no effects were observed in A673 in the same concentration range (Supplementary Figure 3D).

We selected PA4 for the treatment of MNT1 and PA5 for the treatment of SkMel28 and analyzed their effects over time at 1 μM. PA4 significantly decreased MNT1 cell viability at the tested time points. The time response curve of treated MNT1 cells was parallel to the curve of not treated MNT1 cells, suggesting an anti-proliferative effect (Figure 3E). PA5 significantly decreased SkMel28 cell viability at all tested time points and we observed the strongest effect after 72 h, suggesting that a cytotoxic effect occurred after 48 h (Figure 3F).

**Figure 4:**
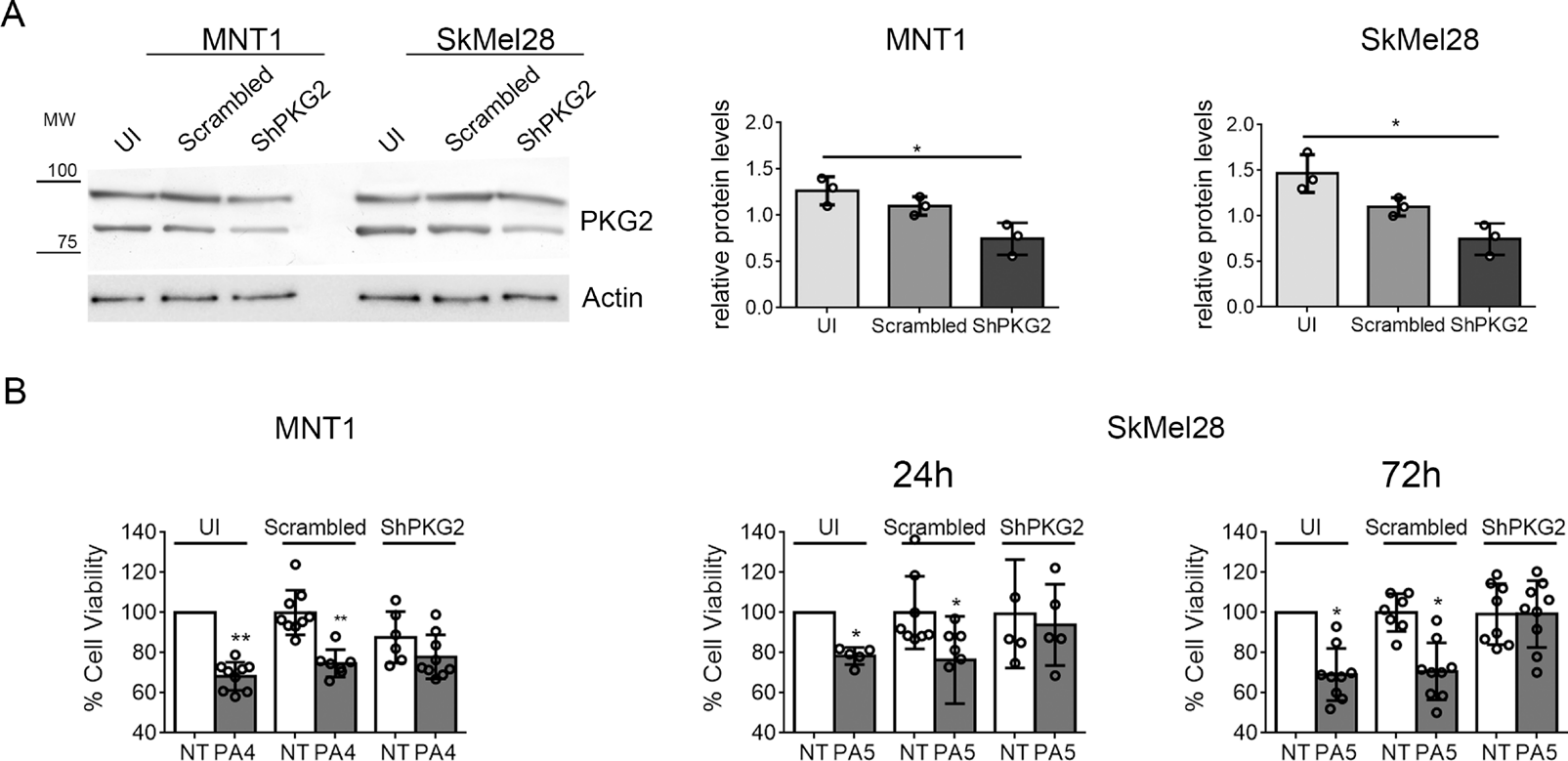
Down-regulation of PKG2 in MNT1 and SkMel28 by shRNA (**A**) Immunoblotting of PKG2 in MNT1 or SkMel28 not infected with retrovirus expressing any shRNA (MNT1-UI and SkMel28-UI), MNT1 or SkMel28 infected with a retrovirus expressing a scrambled control shRNA (MNT1-Scrambled and SkMel28-Scrambled), MNT1 or SkMel28 infected with a retrovirus expressing an shRNA targeting mRNA for PKG2 (MNT1-ShPKG2 and SkMel28-ShPKG2). Western blotting was normalized analyzing actin (lower panel). Histograms at the right-hand side show the quantification of PKG2 protein. (**B**) MTT assay on MNT1-UI, MNT1-Scrambled and MNT1-ShPKG2 not treated (NT) and after treatment with 1 μM of PA4 for 24 h (PA4) and on SkMel28-UI, SkMel28-Scrambled and SkMel28-ShPKG2 not treated (NT) and after treatment with 1 μM of PA5 for 24 h or 72 h (PA5). Not treated cells (NT) in uninfected (UI) samples were set as 100% cell viability. Error bars: SD. Statistical comparison: NT vs treated with Student's unpaired two-tailed *t*-test. Significance levels: *p <* 0.05 = ^*^p < 0.01 = ^**^p < 0.001 =^***^.

In several cancer cell lines reduced viability has been linked to activation of PKG2 [8, 13, 22]. To assess if PA4 and PA5 effects were mediated by PKG2 activation, we downregulated PKG2 expression by a shRNA. Different from a control employing scrambled shRNA, the shRNA targeting *PRKG2* mRNA reduced the PKG2 protein level around ∼30–35% in both cell lines (Figure 4A). MNT1-ShPKG2 and MNT1-scrambled cells were treated with PA4 at 1 μM for 24 h and SkMel28-ShPKG2 and SkMel28-scrambled cells were treated with PA5 at 1 μM for 24 h and 72 h. Reduction of cell viability by PA4 and PA5 were partially lost in PKG2 downregulated cells (Figure 4B), suggesting that the observed reduction of cell viability in MNT1 and SkMel28 treated with PKG activators is, at least partially, mediated by PKG2 activation.

### PKG activators reduce cell proliferation and/or induce cell death of melanoma cells

To understand the specific effect of PA4 and PA5 on MNT1 and SkMel28 cell viability, respectively, cell death was evaluated by ethidium homodimer staining and proliferation by BrdU incorporation.

**Figure 5:**
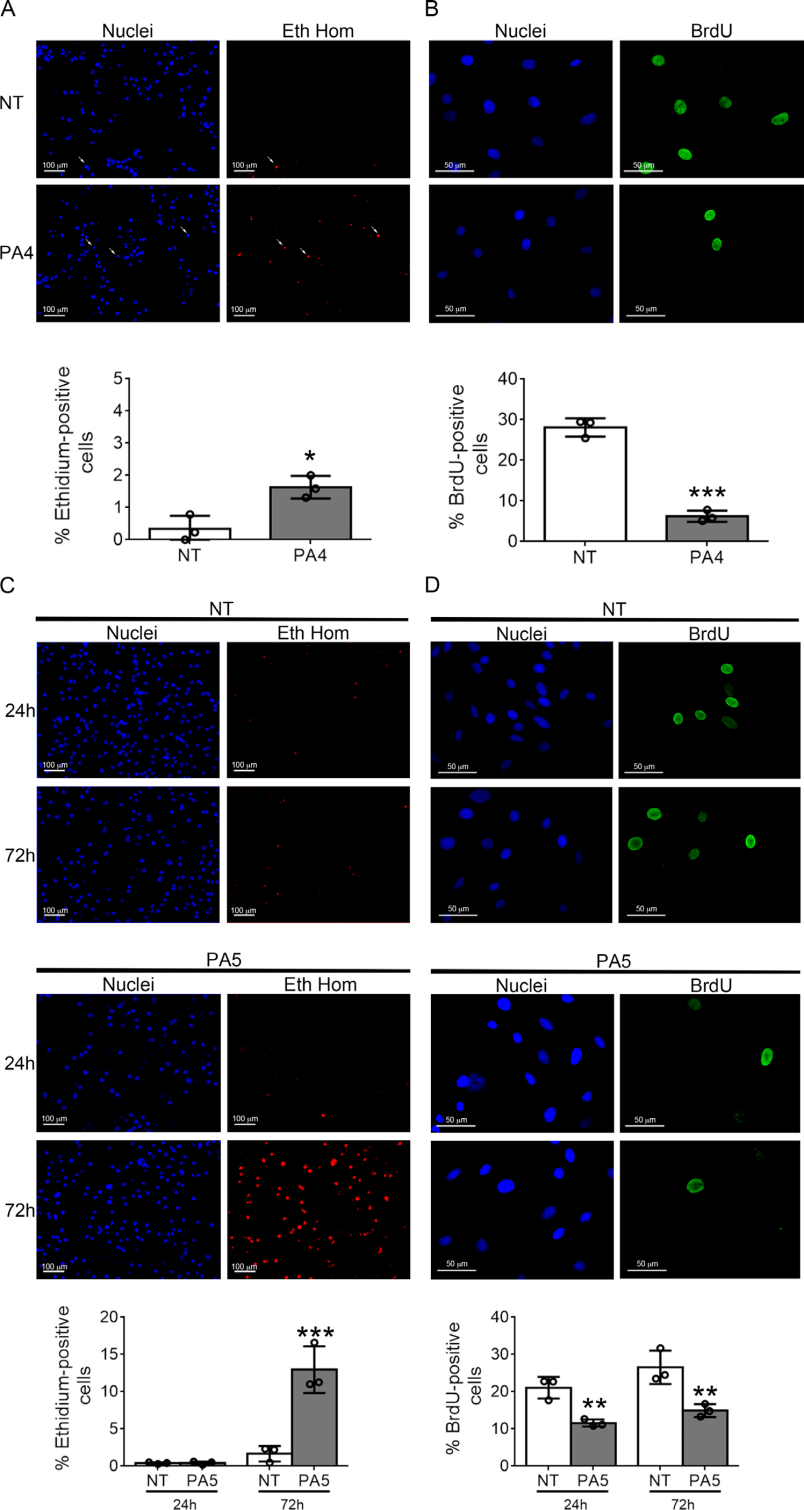
Cell death and proliferation assays (**A**) Cell death was analyzed by ethidium homodimer (Eth Hom) staining in untreated (NT) vs PA4 treated (PA4) MNT1 cells. White arrows indicate positive cells. Percentage of cell death is shown below. (**B**) Cell proliferation was analyzed by BrdU incorporation (BrdU) in untreated (NT) vs PA4 treated (PA4) MNT1cells. Percentage of cell proliferation is shown below. (**C**) Cell death was analyzed by ethidium homodimer (Eth Hom) staining at 24 h and 72 h in untreated (NT) vs PA5 treated (PA5) SkMel28 cells. Percentage of cell death is shown below. (**D**) Cell proliferation was analyzed by BrdU incorporation (BrdU) at 24 h and 72 h in untreated (NT) vs PA5 treated (PA5) SkMel28 cells. Percentage of cell proliferation is shown below. Error bars: SD. Statistical comparison: NT vs treated with Student's unpaired two-tailed *t*-test; Significance levels: *p* < 0.05 = ^*^p < 0.01 = ^**^p < 0.001 = ^***^.

A slight but significant increase of cell death, from 0.8% to 1.6%, was observed in MNT1 cells treated with PA4 for 24 h (Figure 5A). On the other hand, the BrdU incorporation assay showed a strong reduction of cell proliferation from 27% to 7% (Figure 5B) suggesting that PA4 mostly exerts a cytostatic effect on MNT1 cells.

In SkMel28 cells the treatment with PA5 for 24 h did not induce cell death, but after 72 h a strong increase in cell death from 2% to 12% was observed (Figure 5C). Proliferation was reduced from 20% to 11% at 24 h and from 26% to 14% at 72 h (Figure 5D). Therefore, PA5 exerts a cytostatic effect in the first phase of the treatment and a cytotoxic effect is activated after longer time of exposure.

### PA5 reduces motility of SkMel28 cells

**Figure 6:**
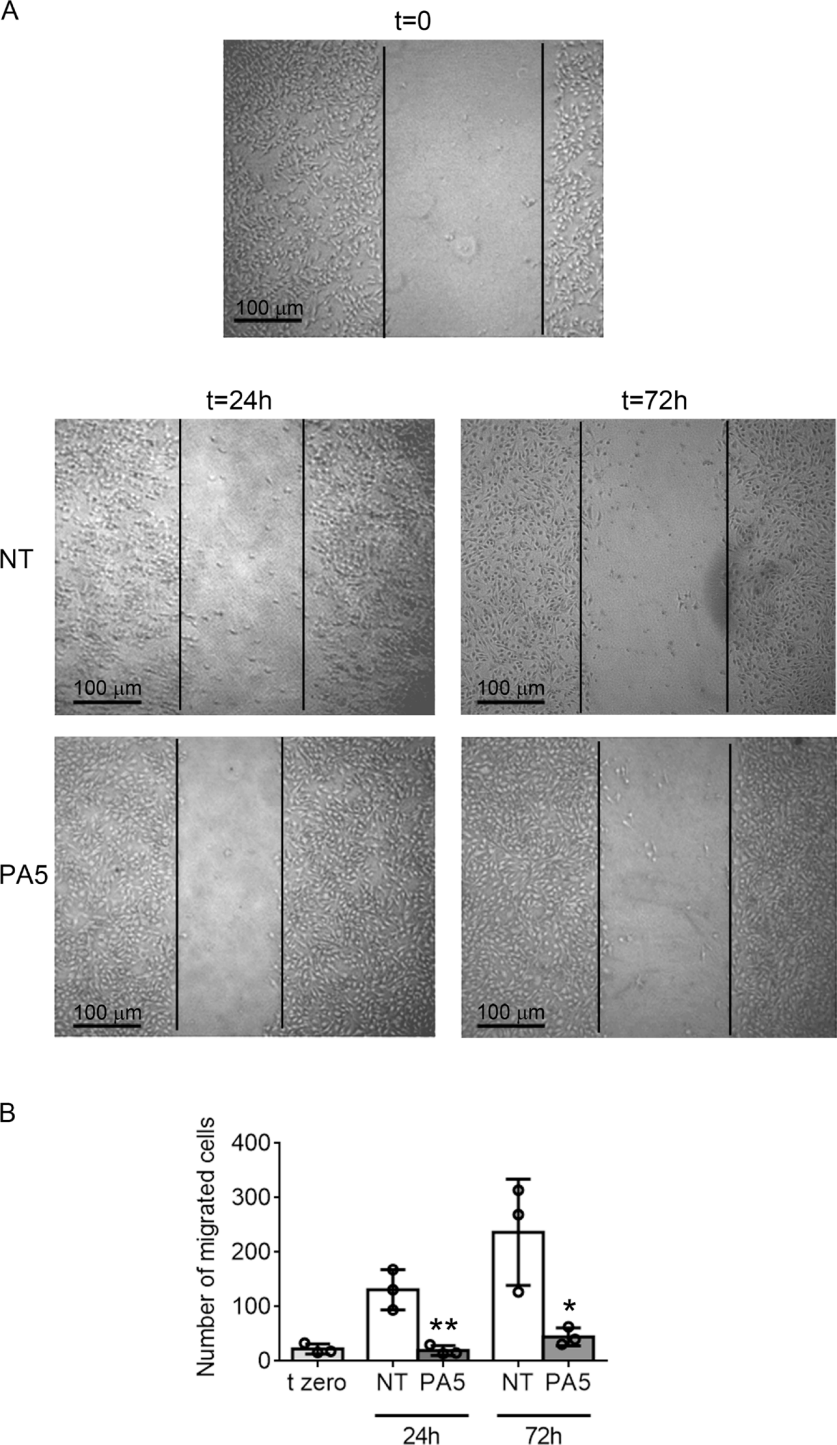
SkMel28 cell migration assay after treatment with PA5 (**A**) Images of the wound healing assay on SkMel28 cells not treated (NT) or treated with 1 μM PA5 for 24 h or 72 h. t = 0 indicates the starting point of the assay. (**B**) The entire wound was analyzed and the number of cells that migrated into the wound was counted. Error bars: SD. Statistical comparison: NT vs treated with Student's unpaired two-tailed *t*-test; Significance levels: *p* < 0.05 = ^*^p < 0.01 = ^**^p < 0.001 = ^***^.

We then examined by *in vitro* wound healing assay whether targeting the cGMP/PKG pathway could also affect cell invasiveness. We performed the test in the absence of serum and in the presence of mitomycin C to block cell proliferation. Mitomycin C did not interfere with cell viability, as shown by MTT assay (Supplementary Figure 4A). While MNT1 cells showed very little migration (Supplementary Figure 4B), SkMel28 cells moved into the scratch with a fibroblast-like migration pattern. Treatment with PA5 significantly affected SkMel28 cell migration (Figure 6A–6B).

### PKG activators modify the phosphorylation status of VASP and RhoA

Cell mobility can be affected by changes in properties of the cytoskeleton. VASP is a protein associated to the cytoskeleton that can be phosphorylated at different sites correlating with different acquired functions into the cells. The main VASP phosphorylation sites are serine 239 (S239) and serine 157 (S157). S239 is preferentially phosphorylated by PKG and is associated with apoptosis, stop of proliferation and stop of migration in different cancer types [27]. S157 is the main target of cAMP-dependent protein kinase (PKA) and has been associated with cell survival and increased motility [27]. We evaluated VASP phosphorylation at S239 and S157 in MNT1 cells after treatment with 1 μM PA4 and in SkMel28 cells after treatment with 1 μM PA5 by immunofluorescence and immunoblotting.

**Figure 7:**
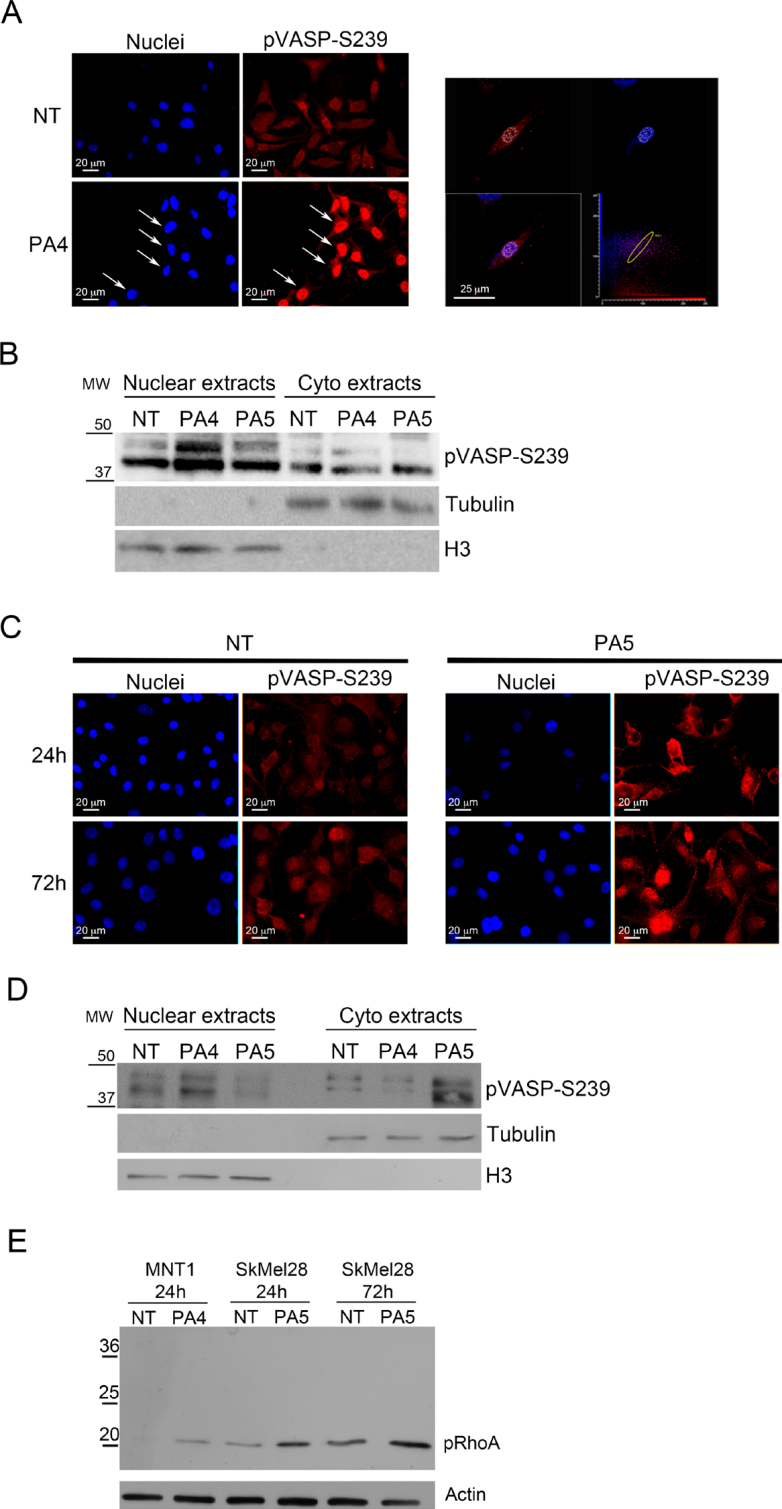
Analysis of VASP phosphorylation at S239 (**A–B**) VASP phosphorylation at S239 was analyzed in MNT1 cells not treated (NT) and treated with 1 μM PA4 for 24 h. (A) Immunolocalization of S239 pVASP shows increased pVASP after treatment. White arrows indicate the nuclear localization of pVASP. At the right-hand side, confocal analysis shows one optical section at the nuclear level confirming nuclear localization of S239 pVASP in PA4 treated MNT1. (B) Immunoblotting on nuclear extracts and cytoplasmic extracts of pVASP phosphorylated at S239 in MNT1 cells not treated (NT) or PA4 and PA5 treated. Treatment was performed at 1μM concentration for 24 h. The blot was normalized by the analysis of tubulin for cytoplasm extracts and histone H3 for nuclear extracts. (**C–D**) VASP phosphorylation at S239 was analyzed in SkMel28 cells not treated (NT) and treated with 1 μM PA5 for 24 h and 72 h. (C) Immunolocalization of S239 pVASP shows increased pVASP after treatment. (D) Immunoblotting on nuclear extracts and cytoplasmic extracts of pVASP phosphorylated at S239 in SkMel28 cells not treated (NT) or PA4 and PA5 treated. Treatment was performed with 1μM concentration of the compounds for 24 h. The blot was normalized by the analysis of tubulin for cytoplasm extracts and histone H3 for nuclear extracts. **(E)** Immunoblotting of RhoA phosphorylated at S188 (pRhoA) in MNT1 cells not treated (NT) or treated with 1 μM PA4 for 24 h and in SkMel28 cells not treated (NT) or treated with 1 μM of PA5 for 24 h or 72 h. The immunoblot was normalized by analysis of actin.

PA4, as expected for a PKG activator, significantly increased S239 phosphorylation after 24 h of treatment in MNT1 cells (Figure 7A–7B) and increased the nuclear localization of pVASP, as demonstrated by confocal analysis (Figure 7A, arrows) and immunoblotting on nuclear extracts (Figure 7B).

In SkMel28 cells PA5 increased pVASP S239 phosphorylation after 24 h and 72 h of treatment (Figure 7C). pVASP phosphorylated at S239 showed a cytoplasmic localization in SkMel28 cells after treatment with PA5 for 24 h, as demonstrated by immunofluorescence (Figure 7C) and immunoblotting on cytoplasmic extracts (Figure 7D).

As control, we treated MNT1 cells with PA5 and SkMel28 cells with PA4 and analyzed S239 pVASP. Major nuclear localization was only detected after treatment with PA4 in both cell lines after 24 h treatment (Figure 7B and 7D; Supplementary Figure 5) suggesting a specific effect of PA4.

We analyzed a second intracellular target of PKG, i.e. RhoA, that can be phosphorylated at S188 by PKG [28]. Treatments with PA4 in MNT1 and with PA5 in SkMel28 induced significant increase of RhoA phosphorylation at S188 (Figure 7E).

### Other potential targets of PA4 and PA5

We demonstrated that PA4 and PA5 activate PKG and that activation of PKG2 in melanoma cells induces a reduction of cell viability. We then assessed whether PA4 and PA5 affected other intracellular targets of cGMP in melanoma cells. We first evaluated possible effects on CNGC that allows calcium ion influx upon cGMP binding. We treated MNT1 and SkMel28 with PA4 and PA5 at 1 μM and analyzed intracellular calcium by staining with Fluo-4 AM 24 h later. MNT1 treated with PA4 showed no significant change in intracellular calcium (Supplementary Figure 6A). Intracellular calcium decreased in SkMel28 treated with PA5 for 24 h and 72 h, suggesting possible binding of PA5 to CNGC with an inhibiting effect.

PDEs are also targets of cGMP and they may hydrolyze the PA compounds. PA4 and PA5 did not affect intracellular cGMP in MNT1 or SkMel28 cells suggesting no inhibiting effect on PDE activity (Supplementary Figure 6B). To assess possible effects of PDEs on PA4 and PA5, we inhibited PDEs by treatment with zaprinast at 0.3 mM. Zaprinast, as expected, increased intracellular cGMP in both cell lines (Supplementary Figure 6B). Viability of both melanoma cell lines was strongly decreased after treatment with PA4 and PA5 in the presence of zaprinast (Supplemantary Figure 6C), possibly due to additive effects of increased cGMP after exposure to the inhibitor. Nevertheless, it cannot be excluded that PA4 and PA5 could be partially degraded by PDEs.

**Figure 8:**
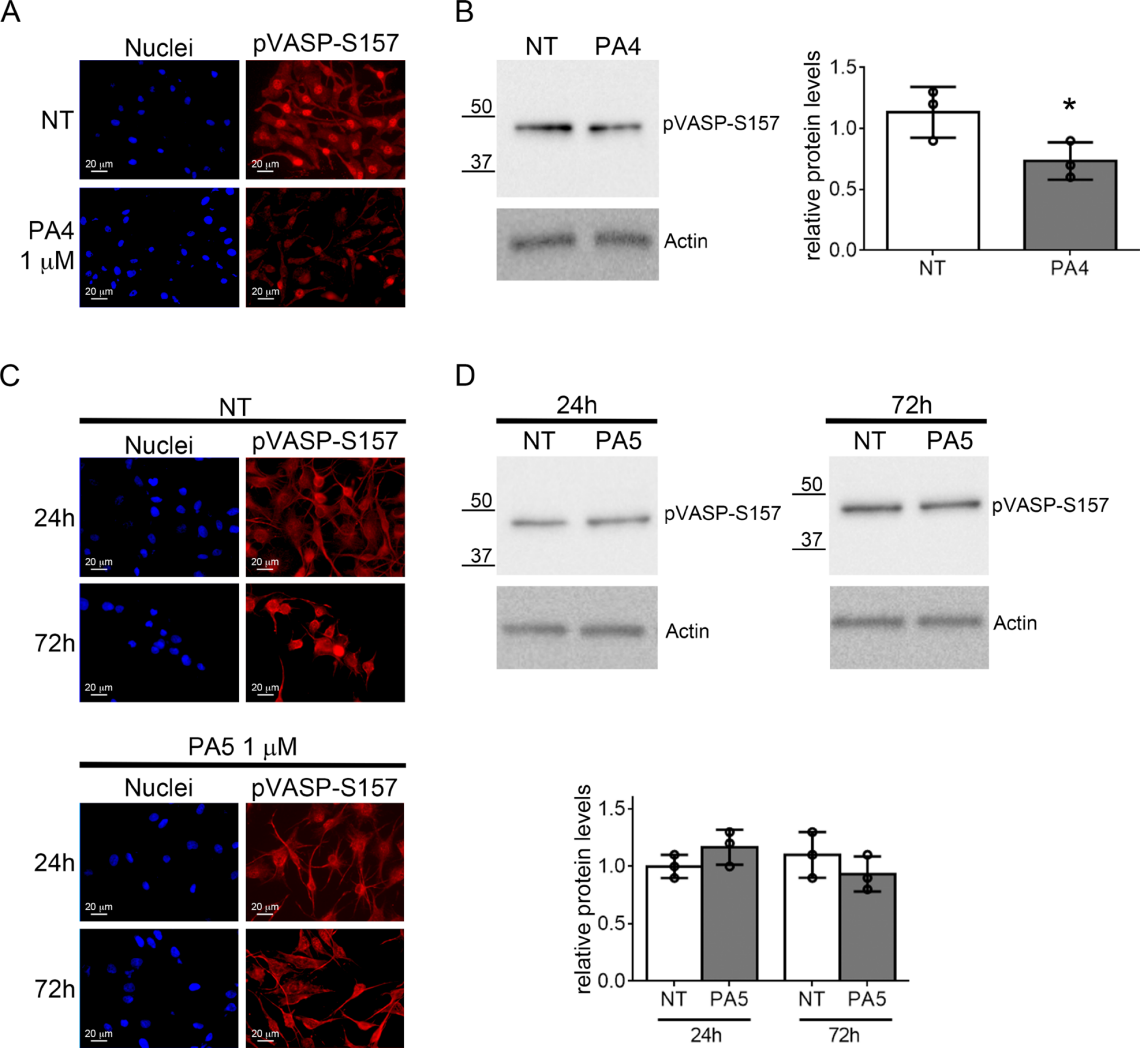
Analysis of VASP phosphorylation at S157 (**A–B**) VASP phosphorylation at S157 was evaluated in MNT1 cells not treated (NT) or treated with PA4 at 1 μM for 24 h. (A) Immunolocalization of S157 pVASP shows decreased pVASP after treatment. (B) Immunoblotting of pVASP phosphorylated at S157 in MNT1 cells. The immunoblot was normalized by analysis of actin. The histogram at the right-hand side shows quantitative analysis of three replicates. (**C–D**) VASP phosphorylation at S157 was evaluated in SkMel28 cells not treated (NT) or treated with PA5 at 1 μM for 24 h or 72 h. (C) Immunolocalization of S157 pVASP shows no change after treatment. (D) Immunoblotting of pVASP at S157 in SkMel28 cells after treatment with PA5 at 1 μM for 24 h or 72 h. The immunoblot was normalized by analysis of actin. The histogram shows quantitative analysis of three replicates. Error bars: SD. Statistical comparison: NT vs treated with Student's unpaired two-tailed *t*-test; Significance levels: *p* < 0.05 = ^*^p < 0.01 = ^**^p < 0.001 = ^***^.

Finally, we evaluated off-target effects of PA4 and PA5 on PKA. We first evaluated the phosphorylation of VASP at S157. S157 pVASP was significantly reduced in MNT1 cells after treatment with 1 μM PA4, as demonstrated by immunofluorescence and immunoblotting analyses (Figure 8A–8B). PA5 at 1 μM was not able to modify the S157 phosphorylation status in SkMel28 cells after 24 h or 72 h (Figure 8C–8D). Based on previous studies with cGMP analogues we expect around 25% of the compound to enter the cells [29]. We thus tested activation of PKA *in vitro* with 250 nM of PA compounds (Table 2). For PKA type Iα we found less than 2% of the cAMP activation for cGMP, PA2 and PA6 whereas PA5 results in 6% activation and PA1 and PA4 around 10% activation. In contrast, for PKA type IIα less than 3% activation could be measured with all compounds in comparison to cAMP activation. We further assessed activation of PKA by treating cells with PA4 and PA5 at 10 μM for 24 h and 72 h (Supplementary Figure 7). No increase of pVASP at S157 was detected in MNT1 but a significant increase was found in SkMel28 after treatment with PA5. Indeed, PA4 and PA5 at 10 μM concentration had 80% and 40% activity compared to cAMP on human type Iα PKA holoenzyme, respectively, and 30% and 10% activity compared to cAMP on human type IIα PKA holoenzyme, respectively. Altogether, these data indicate off-target effects of the compounds on PKA at concentrations higher than those used to decrease cell viability, as also suggested by the loss of effects in the viability assays at high concentrations (see Figure 3C–3D).

**Table 2:** PKA activation

RIα (*n* =4)		RIIα (*n* = 4)	
**basal activity**	0.0 ± 0.8	**basal activity**	0.0 ± 2.1
**cAMP**	100 ± 2	**cAMP**	100 ± 2
**cGMP**	1.7 ± 0.9	**cGMP**	0.6 ± 0.6
**PA1**	10.4 ± 3.3	**PA1**	1.1 ± 2.5
**PA2**	1.4 ± 2.9	**PA2**	0.6 ± 2.2
**PA3**	–0.2 ± 2.2	**PA3**	–0.4 ± 1.5
**PA4**	9.1 ± 0.9	**PA4**	2.2 ± 3.2
**PA5**	6.0 ± 3.1	**PA5**	1.6 ± 3.2
**PA6**	1.6 ± 3.3	**PA6**	1.6 ± 2.6

Normalized PKA activation data of human PKA isoforms RIα and RIIα employing 250 nM of cAMP, cGMP, PA1, PA2, PA3, PA4, PA5 and PA6, respectively, in *in vitro* PKA activation assays (see Materials and Methods). Values denote the mean ± standard deviation (SD). *n* = number of measurements.

## DISCUSSION

Activation of PKG by cGMP analogues is attracting interest in oncology as a new molecular strategy to interfere with tumor progression [3]. Several studies in the last few years have demonstrated the potential application of targeting the cGMP/PKG pathway in the treatment of colon cancer [7, 30, 31] and breast cancers [8, 9, 11] but very little is known about this signaling pathway in melanoma.

Here we synthesized new cGMP analogues with dimeric structures with possibly improved affinity and/or specificity for the different PKG isoforms. These new compounds were designed to activate PKG [32]. The conceptual idea behind PLDs is to achieve a strong enhancement in activity by addressing two binding sites simultaneously with a single molecule. Avidity effects, based on the proximity of a second cGMP unit once the first one is bound to the target, might be of relevance as well. So far, only one homologous series of a PLD and the corresponding impact on PKG1α and CNG channels has been studied [23]. The authors showed a strong activation up to 30-fold for PKG1α, as also found in our *in vitro* activity assays where the dimeric compounds activated at 50–400 times lower concentrations compared to cGMP, and up to 1000-fold for CNG channels, when compared to monomeric cGMP. This class of compounds is thus suggested to be very promising effectors of the cGMP signal transduction cascade. We tested our new PLDs in two melanoma cell lines, i.e. MNT1 and SkMel28, in comparison to three monomeric cGMP analogues. Different effects of the compounds might be associated to the fact that both cell lines expressed PKG1β and PKG2 at similar levels, whereas PKG1α expression was significantly higher in SkMel28. The monomeric cGMP analogues did not show much effect in reducing melanoma cell viability whereas the new dimeric cGMP analogues, i.e. PA4 and PA5, strongly reduced cell viability. This strong effect on cell viability indicates that dimers are cell permeable notwithstanding their complex chemical structures, including two negative charges of their cyclic phosphate units. Based on the fact that PA4 and PA5 increased cell viability in SHSY5Y cells, not expressing PKG2, had no effect on A673 cells, that express only PKG1α, and that the effect is lost upon PKG2 downregulation, it is reasonable to assume that these compounds are able to reduce melanoma cell viability by activating PKG2. On the contrary, activation of the isoforms 1 (1α and/or 1β) promotes cell viability, as suggested by results in SHSY5Y cells. We can hypothesize that in melanoma, similarly to other cancer cell types [8, 13, 22], activation of PKG2 can stop cell proliferation or induce cell death, whereas PKG1 isoforms promote tumor growth as previously reported [15].

Importantly, PA4 and PA5 did not reduce viability of HaCaT, a non-tumor skin keratinocyte cell line expressing all PKG isoforms. We can thus hypothesize that these compounds affect the cGMP/PKG signaling pathway selectively in a tumor context. To date, we can only speculate that the same PKG isoforms may activate separate downstream targets in different cells and this hypothesis needs further and specific investigation.

PA4 and PA5 had mainly a cytostatic effect in melanoma cells with minor cytotoxicity. Only prolonged treatment with PA5 could activate significant cell death in SkMel28. PA5 not only reduced SkMel28 cell viability but also affected cell migration and, interestingly, this correlated with a reduction of intracellular calcium levels. The role of calcium in melanoma cell migration is quite complex and the final effect, i.e. promotion or reduction of motility, depends on the calcium source [33, 34]. Different calcium channels are associated with different and opposite effects but, in general, an increase in intracellular calcium results in enhanced cancer cell migration [33].

Reduced migration in SkMel28 cells by PA5 could be associated to RhoA phosphorylation and to a specific VASP phosphorylation pattern. In fact, cell migration is strongly affected by phosphorylation of VASP [27]. The phosphorylation status of VASP is crucial not only for cell migration but also for cell survival/proliferation or apoptosis [35]. PKA preferentially phosphorylates VASP at S157 promoting F-actin-shaped membrane protrusions [36], while phosphorylation of VASP by PKG at S239 induces dissociation of VASP/actin complexes and inhibition of locomotory membrane structures as well as decreased cell proliferation and increased apoptosis [37–40]. In SkMel28 cells 1 μM PA5 increased S239 pVASP but did not change S157 phosphorylation. Phosphorylation of these two sites are not mutually exclusive and the behavior of the cells depends on the pattern of phosphorylation [27]. Of note is the observation that PA4 caused nuclear localization of phosphorylated VASP and PA5 cytoplasmic localization. It is reasonable to assume that the different effects attributable to pVASP may correlate with different localization of the protein, as previously reported [41, 42], but at the moment, we cannot ascertain whether phosphorylation at S239 happens in the cytoplasm or directly inside the nucleus and we cannot define the role of pVASP in the two cellular compartments. Effects on migration in MNT1 cells could not be tested, but PA4 treatment induced an increase of S239 pVASP with nuclear localization and a decrease in S157 pVASP. This complex pattern of phosphorylation involving also S157 may correlate with the cytostatic and cytotoxic effects of PA4, observed in MNT1 cells. Phosphorylation of RhoA at S188 leading to its inactivation may also contribute to the reduced melanoma cell migration. In fact, RhoA activation was previously linked to global microfilament reorganization and epithelial-to-mesenchymal transition of melanoma cells [43].

Finally, it is important to underline that dimeric cGMP analogues decreased viability of cell lines that differ on the BRAF V600E mutation: homozygosis in SkMel28 and heterozygosis in MNT1. Therefore, targeting the cGMP/PKG pathway could open a new therapeutic strategy for V600E BRAF negative melanoma where no effective treatment is available at the moment.

In conclusion, we developed two new dimeric cGMP analogues able to reduce viability and migration of melanoma cells. Future studies will define the possible use of such cGMP analogues as targeted therapy for melanomas, alone or in combination with standard chemotherapy.

## MATERIALS AND METHODS

### Reagents

Br-PEG_5_-CH_2_CH_2_Br and bis-propargyl-PEG_7_ were purchased from BroadPharm, NH_2_-PEG_n_-(CH_2_)_2_NH_2_ (2000 Da, polydispers) from Iris Biotech, methanol (gradient grade) and acetonitrile (gradient grade) from Honeywell, and ethyl acetate from VWR. All further applied solvents and reagents were obtained from Sigma-Aldrich. Reversed-phase HPLC chromatography was performed on C18 columns using ODS-A-YMC, 120-S-11 material from YMC Europe.

DMEM (Dulbecco's modified Eagle Medium) with high glucose, DMEM:F12, Iscove's medium, AIM-V, BME (Basal Medium Eagle), fetal bovine serum (FBS), phosphate-buffered saline (PBS), penicillin, streptomycin, glutamine, sodium bicarbonate, sodium pyruvate, non-essential amino acids and all other cell culture reagents were purchased from Invitrogen (ThermoFisher Scientific). 3-(4,5 dimethylthiazol-2-yl)-2,5-diphenyltetrazolium bromide (MTT) and bromo-deoxy-uridine (BrdU) were bought from Sigma-Aldrich.

### Synthesis of novel cGMP analogues

#### Synthesis of monomeric precursor and PKG activators (PA)

8- Thioguanosine- 3′, 5′- cyclic monophosphate (8-T-cGMP), β- (4- azidophenyl)- 1, N^2^- etheno- 8- bromoguanosine- 3′, 5′- cyclic monophosphate (4-N_3_-PET-8-Br-cGMP), 8- (4- chlorophenylthio)guanosine- 3′, 5′- cyclic monophosphate (PA1, 8-pCPT-cGMP), 8- bromo- β- phenyl- 1, N^2^- ethenoguanosine- 3′, 5′-cyclic monophosphate (PA2, 8-Br-PET-cGMP), and 8- bromo- (2- naphthyl- 1, N^2^- etheno)guanosine- 3′, 5′- cyclic monophosphate (PA3, 8-Br-(2-N)ET-cGMP) were synthesized in analogy to Sekhar *et al.* [26].

### Synthesis of dimeric PKG activators (PA)

Guanosine- 3′, 5′- cyclic monophosphate- [8- thio- (pentaethoxy)- ethylthio- 8]- guanosine- 3′, 5′- cyclic monophosphate (PA4, cGMP-8-T-(EO)_5_-ET-8-cGMP). *N*,*N*- diisopropylethylamine (17 μl, 102 μmol) and Br-PEG_5_-CH_2_CH_2_Br (9 mg, 23 μmol) were added successively to a solution of 8-T-cGMP (triethylammonium salt, 24 mg, 51 μmol) in dimethyl sulfoxide (DMSO, 400 μl). The reaction mixture was stirred for 4 h. The solvent was removed through high vacuum evaporation with a speedvac concentrator. The residue was dissolved in water (1 ml), washed with ethyl acetate (3x), subjected to preparative reversed phase HPLC and desalted. Yield (purity): 13 μmol, 51% (> 99%). HPLC: (30% MeOH, 10 mM triethylammonium formate (TEAF) buffer, pH 6.8). UV-VIS: **λ**_max_= 274 nm (pH 7), ε = 24660 (est.). ESI-MS (+): *m/z* calculated for C_32_H_46_N_10_O_19_P_2_S_2_Na ([M+Na]^+^): 1023.18, found: 1023. ESI-MS (–): *m/z* calculated for C_32_H_45_N_10_O_19_P_2_S_2_ ([M-H]^-^): 999.18, found: 999.

Guanosine- 3′, 5′- cyclic monophosphate- [8- thiomethylamido- (PEG pd 2000)- amidomethylthio- 8]- guanosine- 3′, 5′- cyclic monophosphate (PA5, cGMP-8-TMAmd-(PEG pd 2000)-AmdMT-8-cGMP). *N*,*N*-diisopropylethylamine (16 μl, 96 μmol) and PyBOP^®^ (26 mg, 50 μmol) were added successively to a solution of 8- carboxymethylthioguanosine- 3′, 5′- cyclic mono-phosphate (8-CMT-cGMP, triethyl ammonium salt, 25 mg, 46 μmol) (See “Supplementary methods” for further details) and NH_2_-PEG_n_-(CH_2_)_2_NH_2_ (2000 Da, polydispers, 44.7 mg, 23 μmol) in DMSO (700 μl). The reaction mixture was stirred for 15 min. Water (100 μl) was added, stirring was continued for 10 min and the solvent was removed through high vacuum evaporation with a speedvac concentrator. The residue was dissolved in water (1 ml), the pH was adjusted to 6 with HCl (1 M) and the solution washed with ethyl acetate (5×). The aqueous phase was evaporated under reduced pressure using a rotary evaporator, redissolved in water, subjected to preparative reversed phase HPLC and desalted, giving the title compound. Yield (purity): 10.8 μmol, 47% (> 95%). HPLC: (Gradient, 21% then 24% MeCN, 50 mM NaH_2_PO_4_ buffer, pH 6.8). UV-VIS: **λ**_max_=275 nm (pH 7), ε = 24660 (est.). ESI-MS (-): *m/z* calculated for C_114_H_206_N_12_O_60_P_2_S_2_ (*n* = 44, [M-2H]^2-^): 1414.62, found: 1414.

8- Bromoguanosine- 3′, 5′-cyclic monophosphate- [1, N^2^-etheno- β- phenyl- 4- yl- (1- [1, 2, 3]- triazole- 4- yl)- methoxy- (hexaethoxy)- methyl- (4- (4- [1, 2, 3]- triazole- 1- yl)- β- phenyl- 1, N^2^-etheno)]- 8- bromoguanosine- 3′, 5′- cyclic monophosphate (PA6, 8-Br-cGMP-ETP-p(1-[1,2,3]- Tz-4)-MeO-(EO)_6_-Me-p(4-[1,2,3]-Tz-1)-PET-cGMP-8-Br). Cu(PPh_3_)_3_Br (0.14 mg, 0.15 μmol) was added to a solution of 4-N_3_-PET-8-Br-cGMP (sodium salt, 18 mg, 31 μmol) and bis-propargyl-PEG_7_ (15 μmol) in water/*N*,*N*-diisopropylethylamine (10:1, v/v, 1 ml) in an amber flask. The reaction mixture was stirred for 16 h. The solvent was removed through high vacuum evaporation with a speedvac concentrator. The residue was dissolved in water (1 ml) and washed with CH_2_Cl_2_ (3 x). The aqueous phase was evaporated under reduced pressure using a rotary evaporator, the residue was redissolved in water, subjected to preparative reversed phase HPLC and desalted, giving the title compound. Yield (Purity): 34% (> 99%). HPLC: (20% MeCN, 50 mM NaH_2_PO_4_ buffer, pH 6.8). UV-VIS: **λ**_max_ =270 nm (pH 7), ε = 72000 (est.). ESI-MS (-): *m/z* calculated for C_54_H_57_Br_2_N_16_O_21_P_2_ ([M-H]^−^): 1485.17, found: 1485.

Dimeric cGMP analogues are patent-pending (Patent application n° EP16186700.7).

### *In vitro* PKG activation assay

FLAG-tagged human PKG1α (2–671) and human PKG1β (5–686) and FLAG-Strep-Strep-tagged human PKG2 [44] were expressed in HEK293T cells. The transfection of the respective constructs was performed on cells at 80% confluency in full medium employing the transfection reagent polyethylenimin (Polysciences Europe GmbH). Cells expressing the FLAG-tagged proteins *h*PKG1α and *h* PKG1β were lysed in PBS buffer (140 mM NaCl, 2.7 mM KCl, 10 mM Na_2_HPO_4_, 1.76 mM KH_2_PO_4_ (pH 7.4)) containing 1% Triton X-100, 1 mM DTT and protease and phosphatase inhibitors (Roche). The proteins were purified using Anti-FLAG-M2 Agarose beads (Sigma-Aldrich) and eluted with PBS buffer containing 5 mg/ml 3xFLAG peptide (Sigma-Aldrich). Cells expressing FLAG-Strep-Strep-tagged *h*PKG2 were lysed using 100 mM Tris-HCl (pH 8), 150 mM NaCl, 1 mM EDTA, 1% Triton X-100 and protease and phosphatase inhibitors (Roche). For purification we employed Strep-Tactin^®^ Superflow^®^ resin (IBA GmbH) and eluted with 100 mM Tris-HCl (pH 8), 150 mM NaCl, 1 mM EDTA and 2.5 mM desthiobiotin (IBA GmbH). The proteins were stored at 4°C in 20 mM MOPS buffer (pH 7) containing 300 mM NaCl and 1 mM DTT.

PKG activities were measured employing microfluidic mobility shift assay applying a Caliper DeskTop Profiler (Caliper Life Sciences, Perkin Elmer). The proteins were incubated in 20 μl buffer (20 mM MOPS (pH 7), 300 mM NaCl, 1 mM DTT, 0.05% L-31, 1 mM ATP, 10 mM MgCl_2_, 0.1 mg/ml BSA, 990 μM Kemptide and 10 μM fluorescein isothiocyanate Kemptide (Biaffin GmbH & Co KG) and various concentrations of the respective cyclic nucleotides at room temperature in a 384-well plate (Corning LV, nonbinding surface) for 2 – 4 h to achieve a substrate conversion of at least 5%. As a control, reaction mixtures without cyclic nucleotides were used. Substrate and product were electrophoretically separated using a ProfilerPro LabChip (4-sipper mode; Perkin Elmer) employing the following conditions: downstream voltage: –150 V, upstream voltage: –1800 V, screening pressure: –1.7 psi. Substrate conversion was plotted against the logarithmic cyclic nucleotide concentration. Activations constants (*K*_act_) were calculated from sigmoidal dose-response curves using GraphPad Prism 6.01. The measured *K*_act_ suggest that the assays were performed on dimeric PKG [45]. Two independent protein preparations were used for each assay.

### *In vitro* PKA activation assay

Recombinant human PKA Cα1 catalytic subunit was expressed and purified as described previously [46, 47]. Recombinant human PKA regulatory subunits (RIα, RIIα) were expressed and purified according to the procedure of Bertinetti *et al.* [48] using Sp-8-AEA-cAMPS agarose. SDS-PAGE was used to monitor protein expression and purification. The recombinant proteins were purified to >95% homogeneity.

PKA holoenzymes were formed by mixing the R- and C-subunit at a molar ratio of 1.2:1 and extensive dialysis against 20 mM MOPS pH 7.0, 150 mM NaCl, 2.5 mM β-mercaptoethanol, supplemented with 1 mM ATP and 10 mM MgCl_2_ for PKA-type I isoform at 4°C overnight.

PKA activity was assayed *in vitro* using the coupled spectrophotometric assay described by Cook *et al.* [49]. Kinase activity was determined after preincubating 30 nM holoenzyme for 1 minute with 250 nM of the respective cyclic nucleotide in the assay mixture comprising 100 mM MOPS (pH 7), 10 mM MgCl_2_, 100 mM ATP, 1 mM phosphoenolpyruvate, 15 U/ml lactate dehydrogenase, 70 U/ml pyruvate kinase, 200 mM reduced nicotinamide adenine dinucleotide and 5 mM β-mercaptoethanol. The kinase reaction was started with 260 μM Kemptide (LRRASLG; GeneCust, Dudelange, Luxembourg) as a PKA substrate. Two independent protein preparations were used for each assay. Data were analyzed using GraphPad Prism 6.01 (GraphPad Software, Inc, La Jolla, CA, USA) by calculating the normalized activation (respective cAMP activation was set to 100% and the basal activity without cyclic nucleotide to 0%). Normalized data of all measurements (*n* = 4) are shown with SD.

### Cell culture and treatments

Two human malignant melanoma cell lines, MNT1 and SkMel28 (ATCC^®^ HTB-72™), one non-tumor cell line derived from human keratinocytes, HaCaT (CLS-cod 300493), a human neuroblastoma cell line, SHSY5Y (ATCC^®^ CRL-2266^TM^) and an Ewing's sarcoma cell line, A673 (ATCC^®^ CRL-1598^TM^) were used. MNT1 cells were grown in DMEM high glucose (4.5 mg/ml) supplemented with 10% v/v AIM-V, 20% v/v FBS, 2 mM glutamine, 1 mM sodium pyruvate, 0.1 mM non-essential amino acids, 100 U/ml penicillin and 100 μg/ml streptomycin as in [50]. SkMel28 cells, kindly obtained from Prof. Alessandra Marconi, were cultured in BME supplemented with 10% FBS, 1 mM glutamine, 1 mM sodium pyruvate, 2 mM sodium bicarbonate, 0.15 mM non-essential amino acids, 100 U/ml penicillin and 100 μg/ml streptomycin. HaCaT cells, a gift from Dott. Alessandra Recchia, were cultured in DMEM high glucose (4.5 mg/ml) supplemented with 10% FBS, 2 mM glutamine, 100 U/ml penicillin and 100 μg/ml streptomycin. SHSY5Y cells, kindly obtained from Prof. Massimo Dominici, were grown in DMEM:F12 supplemented with 10% FBS, 2 mM glutamine, 100 U/ml penicillin and 100 μg/ml streptomycin. A673, kindly obtained from Prof. Massimo Dominici, were cultured in Iscove's medium supplemented with 10% FBS, 2 mM glutamine, 100 U/ml penicillin and 100 μg/ml streptomycin. Cells were maintained at 37°C in a humidified atmosphere of 5% CO_2_ and sub-cultured every 2–3 days.

For MTT assays, 5 × 10^3^ cells/well were plated into 96-well culture plates in complete medium for 6–8 h to allow cell attachment and then the medium was changed with serum-free medium. In dose-response experiments, 18 h after culture in serum-free medium, cells were exposed for 24 h to various concentrations (from 100 μM to 1 nM) of test compounds in serum-free medium.

For cell death, proliferation and Fluo-4 AM assays, 5 × 10^4^ cells/well were plated on glass slides into 24-well culture plates in complete medium for 6–8 h and the medium was then changed with serum-free medium. Cells were treated for 24 h with serum-free medium containing 1 μM PA4 or 1 μM PA5. SkMel28 cell were also treated with 1 μM PA5 for 72 h replacing the compound every day.

### RT-PCR

Total RNA was extracted from the cells by RNeasy Mini Kit (Qiagen) following the manufacture's instruction, and the purity and integrity were determined by A260/A280 ratio and agarose gel analysis. Total RNA, 1 μg for each sample, was reverse transcribed using the Transcriptor First Strand cDNA Synthesis Kit (Roche) according to the manufacturer's protocol.

PCR was performed with 2 μl of cDNA using specific primers for *PRKG1* variant α and β, *PRKG2* and *S26* (Table 3). Amplified products were analyzed by electrophoresis in a 3% agarose gel and visualized by Ethidium Bromide staining.

**Table 3:** Primers for RT-PCR

	Forward primer	Reverse primer
**PKG1α (100 bp)**	5′-GACAGGCATTCCGGAAGTTC-3′	5′-TGCGACAGCTCCAAGTTCTT-3′
**PKG1β (119 bp)**	5′-CCAGGATCTCAGCCATGTG-3′	5′-GGATCTGCGACAGCTCCA-3′
**PKG2-Variant1 (397 bp)**	5′-TACCTTGAAGGATATGTGGCAAACC-3′	5′-GCCTCCAGAAGCATGTATACATAC-3′
**PKG2-Variant 6 (310 bp)**		
**S26 (138 bp)**	5′-CCGTGCCTCCAAGATGACAA-3′	5′-CGAATGACGAATTTCTTAATGGCC-3′

In brackets the length in base pairs (bp) is indicated.

### Western blot analysis

Total proteins were extracted from cells by lysis in RIPA extraction buffer (50 mM Tris.HCl pH 7.5, 150 mM NaCl, 1 mM EDTA, 1% NP-40, 0.1% w/v SDS, 1 mM Na_3_VO_4_, 1 mM NaPO_4_, 1X protease inhibitor cocktail Sigma-Aldrich). Chromatin was shredded by passing the lysate through a syringe needle (gauche 20). The soluble protein fraction was harvested by centrifugation at 17000 x g for 15 min at 4°C after incubation for 1 h at 4°C in rotation. Protein concentration was determined by Bradford assay (Bio-Rad).

For the enriched nuclear protein fraction the harvested cells were transferred to a 2ml Dounce homogenizer with 200 ml of cold homogenizing buffer (20 mM HEPES.KOH pH 7.5, 250 mM sucrose, 10 mM KCl, 1.5 mM MgCl_2_, 1 mM EDTA, 1 mM EGTA, 1 mM DTT, 1 X protease inhibitor cocktail by Sigma-Aldrich, 1 mM Na_3_VO_4_, 1 mM NaPO_4_) and incubated 30 minutes in ice. Cells were disrupted with 40 strokes and centrifuged at 900 × g for 5 minutes at 4°C. Precipitated nuclei were lysed in RIPA buffer as described above and the supernatant saved as cytosolic protein fraction. Protein concentration was determined by Bradford assay (Bio-Rad).

Proteins were separated by SDS-PAGE in 8% or 10% gels for analysis of PKG2 and PKG1, respectively, and transferred to PVDF membranes (Amersham, GE Healthcare Life Sciences). The membranes were blocked in 5% BSA (PKG1α, PKG1β, pVASP at S239, pVASP at S157 and pRhoA at S188) or 5% non-fat dried milk (PKG2, actin, tubulin and histone H3) for 1 h at room temperature and incubated with the primary antibodies, as reported in Table 4, overnight at 4°C. Horseradish peroxidase-conjugated anti-rabbit (1:10000), anti-mouse (1:5000) (Amersham, GE Healthcare Life Sciences) or anti-goat (1:25000) (Santa Cruz Biotechnologies) secondary antibodies were incubated for 1 h at room temperature. Proteins were visualized using Femto Kit (Euroclone) for PKG1α and PKG1β, Clarity Kit (Bio-Rad) for PKG2, pVASP at S239, pVASP at S157 and pRhoA at S188, and Pico Kit (Euroclone) for actin, tubulin and histone H3. Quantification was performed using the ChemiDoc Imaging System (Bio-Rad).

**Table 4:** List of primary antibodies used in western blotting (WB) and immunofluorescence (IF) experiments

Antibody	Provider	Cat. no.	Dilution	Application
Goat-anti-PKG1α	Santa Cruz Biotechnology	Sc-10335	1:1000 1:100	WB IF
Goat-anti-PKG1β	Santa Cruz Biotechnology	Sc-10341	1:1000 1:100	WB IF
Rabbit-anti-PKG2	Santa Cruz Biotechnology	Sc-25430	1:1000 1:100	WB IF
Goat-anti-BIP (GRP78 (c-20)	Santa Cruz Biotechnology	Sc-1051	1:100	IF
Mouse-anti-cytochrome C	BD Biosciences	556432	1:100	IF
Sheep-anti-cGMP	Jan de Vente & Harry Steinbusch, Maastricht University, the Netherlands	///	1:500	IF
Mouse anti-pVASP at S239	Nanotools	0047-100/VASP-16C2	1:2500 1:250	WB IF
Mouse anti-pVASP at S157	Nanotools	0085-100/VASP-5C6	1:2500 1:250	WB IF
Rabbit anti-pRhoA at S188	Abcam	Ab32046	1:1000	WB
Mouse-anti-actin	Sigma-Aldrich	A2228	1:2000	WB
Rabbit anti-Histone H3	Cell Signaling	9715	1:1000	WB
Mouse anti- Tubulin	Cell Signaling	2144	1:2000	WB

### Immunofluorescence

Cells were fixed with 4% paraformaldehyde (PFA) in PBS at room temperature for 10 min and washed three times in PBS. MNT1 cells were incubated with 30 mM NH_4_Cl at room temperature for 30 min and blocked in blocking buffer (3% BSA, 0.2% Triton-X100 in PBS for the PKG isoforms; 3% BSA in 50 mM Tris-Cl pH 7.5, 150 mM NaCl, 0.2% v/v Tween20 for pVASP at S239 and pVASP at S157) for 45 min. Slides were incubated overnight at 4°C with primary antibodies as listed in Table 4. After incubation for 45 min with Alexa Fluor 568 or Oregon Green anti-rabbit, anti-goat, anti-mouse or anti-sheep secondary antibodies (1:1000, Molecular Probes), the nuclei were stained with 0.0005% DAPI (4′,6-diamidino-2-phenylindole) (Molecular Probes) and slides were cover-slipped with Mowiol (Sigma) and photographed using a Zeiss Axioskop 40 fluorescence microscope (Zeiss) or a confocal microscope Leica TCS-SP2 (Leica).

### Down-regulation of PKG2 by shRNA

A sequence beginning with an AA dinucleotide was chosen in the *PRKG2* mRNA, and we designed hairpin RNAi template oligonucleotides (shRNA). The control shRNA was generated from a scrambled sequence not targeting any known gene. The sequences are as following: *PRKG2*: AAGTGGAATACTATGACAAAG, Scrambled: GCATACTACCACTAGAGTTTA.

Retroviruses from pSUPER.retro.puro vector (OligoEngine, Seattle, WA, USA), expressing the shRNAs, were prepared by transient transfection in amphotropic Phoenix-AMPHO packaging cells (ATCC, Rockville, MD, USA) as in [51]. MNT1 and SkMel28 cells were infected with either PKG2 or scramble shRNA expressing retroviruses. Downregulation of PKG2 protein on MNT1-ShPKG2, MNT1-Scrambled, SkMel28-ShPKG2 and SkMel28-Scrambled was assessed by immunoblotting.

### Cell viability by MTT assay

Cell viability was determined by a colorimetric MTT assay incubating cells with 50 μl of 1 mg/ml MTT aqueous solution for 90 min at 37°C. The supernatant was carefully removed, and the resulting purple formazan crystals were dissolved in 100 μl of isopropanol. The plates were shaken for 10 min and the absorbance was measured at 570 nm using a microplate reader (Labsystems Multiskan MCC/340). The absorbance of untreated cells in each experiment was set to 100% and the percentage of cell viability of treated cells was consequently calculated.

In time-course experiments, after 18 h without serum (time 0), the cells were treated with 1 μM of PA4 or 1 μM of PA5 every 24 h for 3 days and cell viability was assessed at time points: 0 h, 24 h, 48 h and 72 h. The absorbance of untreated cells at time point 0 in each experiment was set to 100% and the percentage of cell viability of treated cells was consequently calculated.

### Cell death assay by ethidium homodimer staining

At the fixed end-point the medium was removed and the cells were fixed in 4% PFA in PBS for 10 min at room temperature and washed with PBS. MNT1 cells were incubated with 30 mM NH_4_Cl for 30 min at room temperature. Slides were incubated in the dark with 1 μM Ethidium Homodimer-1 (Molecular Probes) for 2 min and washed with PBS. Nuclei were stained in blue with 0.0005% DAPI and slides were cover-slipped with Mowiol and photographed using a Zeiss Axioskop 40 fluorescence microscope. Percentages of dying cells were calculated in at least three independent biological replicates.

### Proliferation assay by BrdU incorporation

At the fixed end-point the medium was replaced with medium containing 1 μM BrdU and incubated for 3 h at 37°C. The cells were washed with PBS and fixed with 4% PFA in PBS for 10 min at room temperature. After washing with PBS cells were incubated with 2 N HCl for 30 min at 37°C and the neutral pH was restored by incubation with 0.1 M borate buffer pH 8.5 for 15 min at room temperature.

Slides were washed with PBS and blocked with 5% BSA and 0.3% Triton-X100 in PBS for 1 h at room temperature. The mouse-anti-BrdU (1:100, Developmental Hybridoma) primary antibodies were incubated overnight. After 1 h incubation with Oregon Green-anti-mouse secondary antibodies (1:1000, Molecular Probes) in the presence of 0.0005% DAPI, slides were cover-slipped with Mowiol and photographed using a Zeiss Axioskop 40 fluorescence microscope. Percentages of proliferating cells were calculated in at least three independent biological replicates.

### Wound healing assay

SkMel28 cells were seeded on plates (diameter 3 cm) and grown until confluence. The medium was replaced with serum-free medium for 18 h and confluent cells were treated with 1 μg/ml mitomycin C and wounded with a 1 mm linear scratch by a sterile pipette tip. One hour before wounding, cells were treated with 1 μM PA5 and the compound was maintained during the entire assay. Images were taken at 0 h, 24 h, 48 h and 72 h after wounding using an invertoscope (Leica) equipped with a CCD camera. The entire wound area was examined and the cells counted.

### Calcium imaging by Fluo-4 AM assay

At the fixed end-point Ca^2+^ ion measurements were performed by Fluo-4 AM (Life Technologies) staining. The medium was removed and the cells were incubated with Fluo-4 AM for 30 min at 37°C in calcium- free medium. Cells were fixed with 4% paraformaldehyde for 10 min at room temperature; MNT1 were incubated with 30 mM NH_4_Cl for 30 minutes at room temperature and nuclei were stained with DAPI. Cells were analyzed with a Zeiss Axioskop 40 fluorescence microscope. The fluorescence intensity of each cell was measured with ImageJ software from at least 20 cells in at least three biological replicates.

### Statistical analysis

All assays were repeated at least three times. The results of quantitative studies are reported as mean ± SD. Data from untreated and treated samples were compared by unpaired Student's *t*-test. Paired *t*-test was used to compare untreated and treated samples in time-course experiments. In all cases *p*-value (P) of less than 0.05 was considered significant.

## SUPPLEMENTARY MATERIALS FIGURES AND TABLE


