# Supplementary material for: New cGMP analogues restrain proliferation and migration of melanoma cells
Source: Oncotarget. 2017 Dec 25;9(4):5301–20. doi: 10.18632/oncotarget.23685 (PMC5797051; doi:10.18632/oncotarget.23685)
Supplement: Supplementary file 1 [file oncotarget-09-5301-s001.pdf]

New cGMP analogues restrain proliferation and migration of melanoma cells

SUPPLEMENTARY MATERIALS

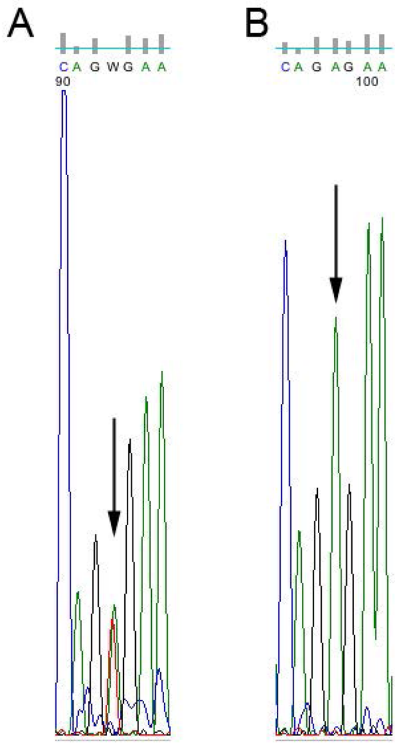

Supplementary Figure 1: BRAF V600E (T>A) mutation in (**A**) MNT1 and (**B**) SkMel28.

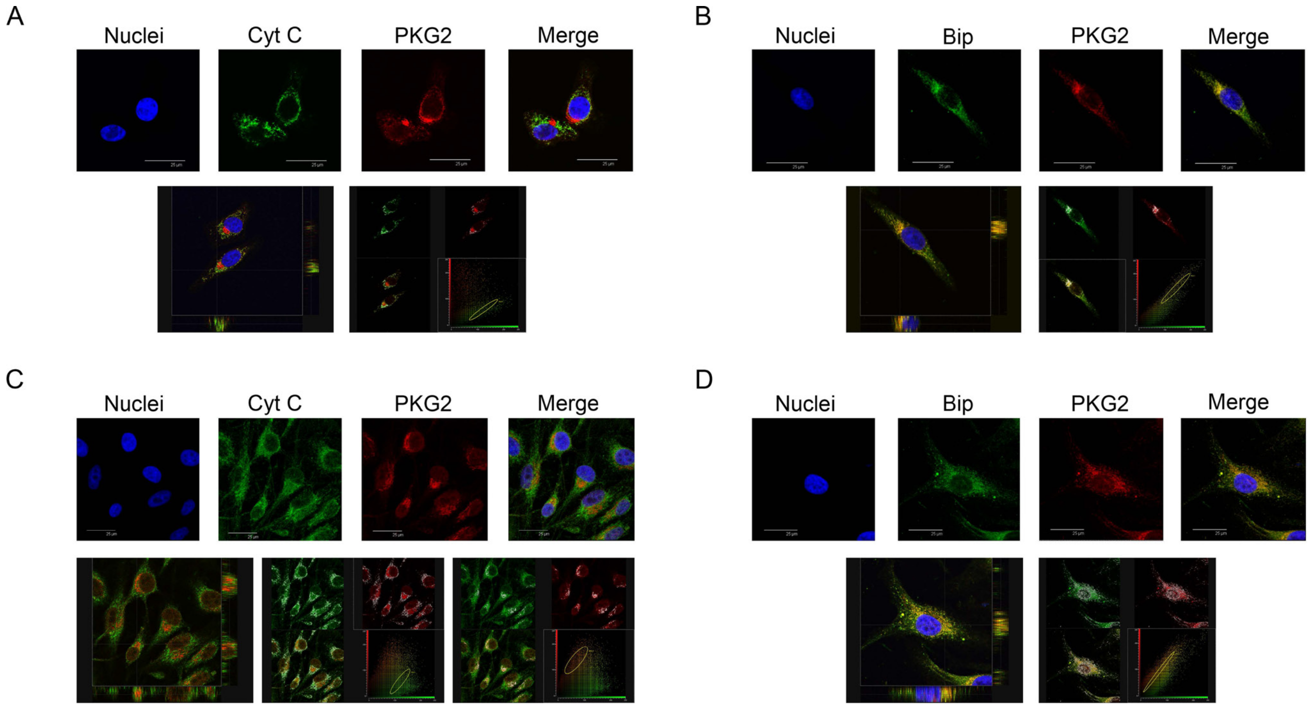

Supplementary Figure 2: Confocal images of intracellular PKG2 localization. (**A**) Double staining of Cytochrome C (Cyt C; green), labeling mitochondria, and PKG2 (red) in MNT1 cells. Lower panels show the analysis at the confocal microscope confirming no co-localization of PKG2 with mitochondria membranes. (**B**) Double staining of Bip (green), an ER resident protein, and PKG2 (red) in MNT1 cells. Lower panels show the analysis at the confocal microscope demonstrating strong co-localization (yellow) of PKG2 with ER membranes. (**C**) Double staining of Cytochrome C (Cyt C; green) and PKG2 (red) in SkMel28 cells. Lower panels show the analysis at the confocal microscope demonstrating partial co-localization (yellow) of PKG2 with mitochondria membranes. (**D**) Double staining of Bip (green) and PKG2 (red) in SkMel28 cells. Lower panels show the analysis at the confocal microscope demonstrating strong co-localization (yellow) of PKG2 with ER membranes.

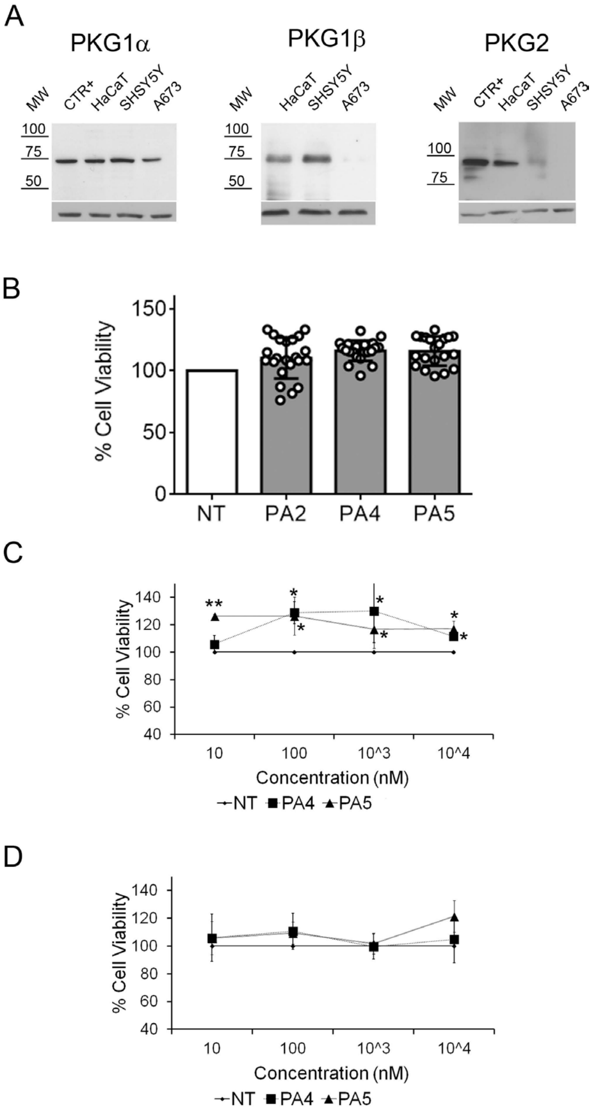

Supplementary Figure 3: (**A**) PKG isozyme expression in HaCaT, SHSY5Y and A673 cells as assessed by immunoblotting. MW = Molecular weight indicated as kDa. 661W cells were used as positive control for PKG1α, SHSY5Y cells were the positive control for PKG1β and CaCo2 colon cancer cells were used as positive control for PKG2. Actin (shown in lower panels) was used as loading control. (**B**) Cell viability assay on HaCaT cells. The cells were treated with monomer PA2 at 1 μM and dimers PA4 and PA5 at 1 μM for 24 h. Values of not treated cells (NT) were set as 100% cell viability. Error bars: SD. Statistical comparison: NT vs treated for each compound with Student's unpaired two-tailed *t*-test. (**C**) Cell viability assay of SHSY5Y cells. Cells were treated with PA4 and PA5 in a range from 10 nM to 10 μM. Values of not treated cells (NT) were set as 100% cell viability. (**D**) Cell viability assay of A673 cells. Cells were treated with PA4 and PA5 in a range from 10 nM to 10 μM. Values of not treated cells (NT) were set as 100% cell viability. Error bars: SD. Statistical comparison: NT vs treated at each concentration for each compound with Student's unpaired two-tailed *t*-test. Significance levels: $p < 0.05 = {}^{*}p < 0.01 = {}^{**}p < 0.001 = {}^{***}$.

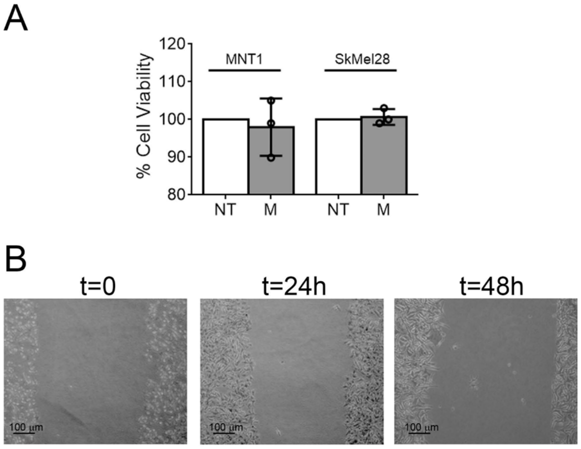

Supplementary Figure 4: (**A**) MTT assay on MNT1 and SkMel28 cells not treated (NT) or treated with 1 ng/ml of mitomycin C for 24 h (M). Not treated cells were set equal to 100% of cell viability. Error bars: SD. Statistical comparison: NT vs treated with Student's unpaired two-tailed *t*-test; $p < 0.05 = {}^{*}p < 0.01 = {}^{**}p < 0.001 = {}^{***}$. (**B**) Wound healing assay on not treated MNT1 cells. MNT1 cells did not move into the lesion in the absence of serum and in presence of 1 ng/ml of mitomycin C.

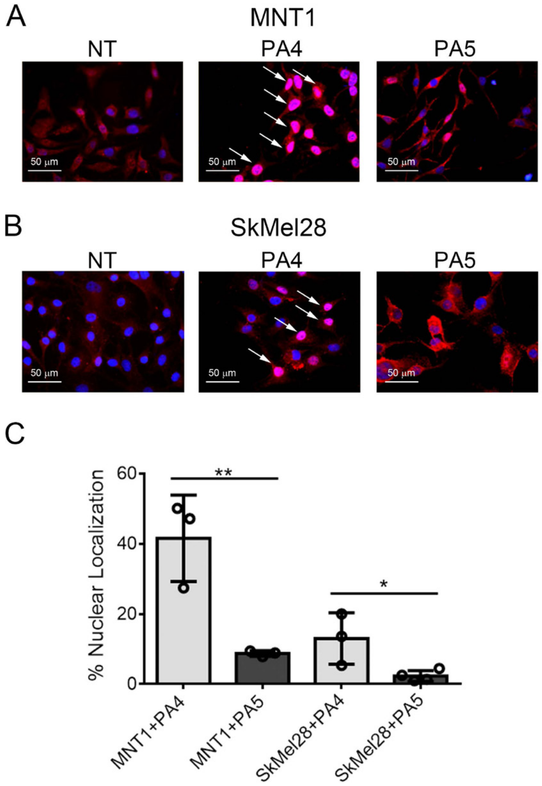

Supplementary Figure 5: VASP phosphorylation at S239 in MNT1 cells (**A**) and SkMel28 cells (**B**) not treated (NT) or treated with 1 µM PA4 and 1 µM PA5 for 24 h. White arrows indicate nuclear localization of pVASP. (**C**) Histogram showing the percentages of cells with nuclear localization of pVASP. Error bars: SD. Statistical comparison: NT vs treated with Student's unpaired two-tailed *t*-test; $p < 0.05 = {}^{*}p < 0.01 = {}^{**}p < 0.001 = {}^{***}$.

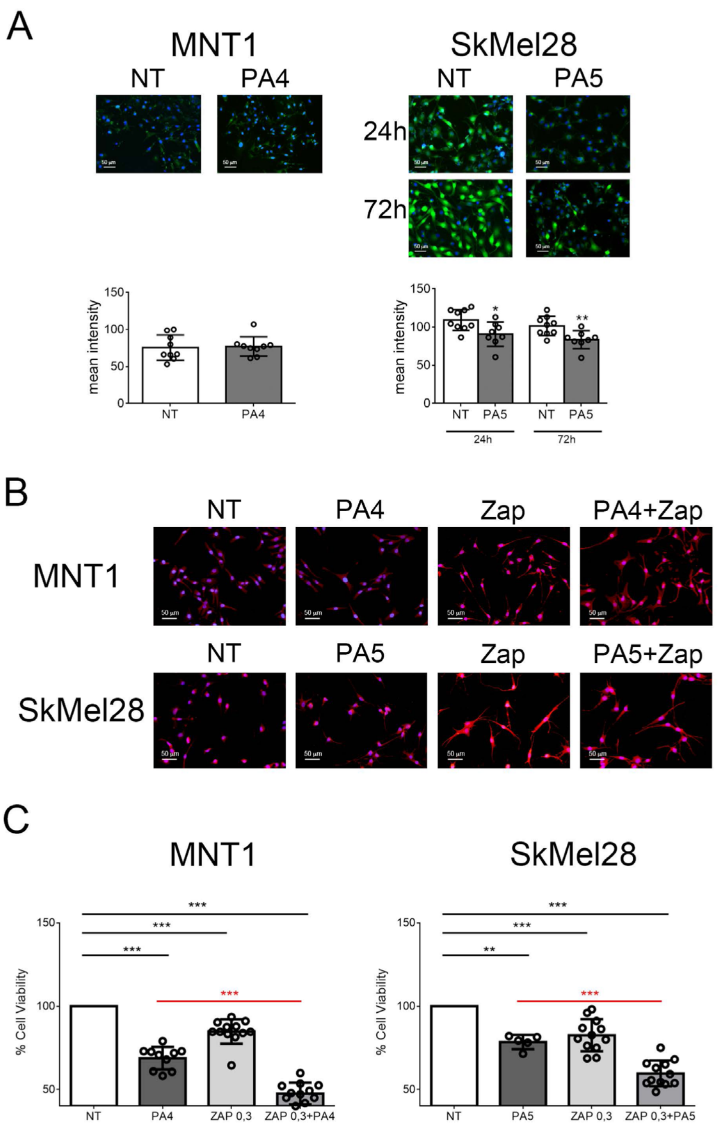

Supplementary Figure 6: (**A**) Calcium analysis, as assessed by Fluo-4 AM staining, in MNT1 cells (on the left-hand side) and in SkMel28 cells (on the right-hand side) not treated (NT) or treated for 24 h with PA4 at 1 µM or PA5 at 1 µM for 24 h and 72 h, respectively. The histograms show the mean intensities of fluorescence of Fluo-4 AM staining in the entire cell as measured by Image J ($N = 10$ cells). (**B**) Upper panel: immunofluorescence of cGMP in MNT1 cells, not treated (NT), treated with PA4 at 1 µM, or with Zaprinast at 0.3 mM (Zap) or with both PA4 at 1 µM and Zaprinast at 0.3 mM (PA4+Zap). Lower panel: immunofluorescence of cGMP in SkMel28 cells, not treated (NT), treated with PA5 at 1 µM, or with Zaprinast at 0.3 mM (Zap) or with both PA5 at 1 µM and Zaprinast at 0.3 mM (PA5+Zap). (**C**) MTT assay in MNT1 cells (on the left-hand side) and SkMel28 cells (on the right-hand side). MNT1 cells were treated with PA4 at 1 µM, or with Zaprinast at 0.3 mM (ZAP 0.3) or with both PA4 at 1 µM and Zaprinast at 0.3 mM (ZAP 0.3+PA4) for 24 h. Not treated cells were set equal to 100% of cell viability. SkMel28 cells were treated with PA5 at 1 µM, or with Zaprinast at 0.3 mM (Zap 0.3) or with both PA5 at 1 µM and Zaprinast at 0.3 mM (ZAP 0.3+PA5) for 24 h. Not treated cells were set equal to 100% of cell viability. Error bars: SD. Statistical comparison: NT vs treated with Student's unpaired two-tailed *t*-test; $p < 0.05 = {}^{*}p < 0.01 = {}^{**}p < 0.001 = {}^{***}$.

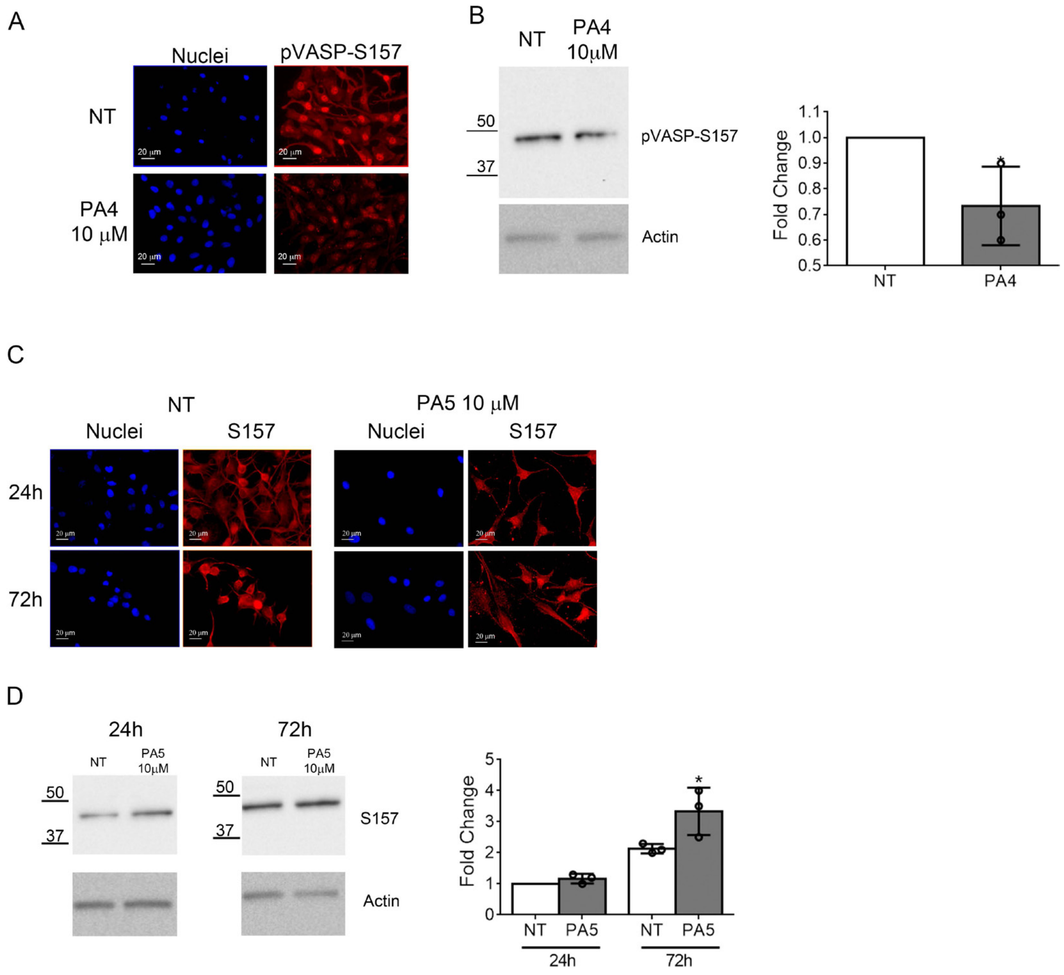

Supplementary Figure 7: Analysis of VASP phosphorylation at S157. (**A**) VASP phosphorylation at S157 was evaluated by immunofluorescence in MNT1 cells not treated (NT) or treated with PA4 at 10 μM for 24 h. (**B**) Immunoblotting of pVASP phosphorylated at S157 in MNT1 cells. The immuoblot was normalized by analysis of actin. The histogram shows quantitative analysis of three replicates. (**C**) VASP phosphorylation at S157 was evaluated by immunofluorescence in SkMel28 cells not treated (NT) or treated with PA5 at 10 μM for 24 h and 72 h. (**D**) Immunoblotting of pVASP phosphorylated at S157 in SkMel28 cells. The immunoblot was normalized by analysis of actin. The histogram shows quantitative analysis of three replicates. Error bars: SD. Statistical comparison: NT vs treated with Student's unpaired two-tailed *t*-test; Significance levels: $p < 0.05 = {}^{*}p < 0.01 = {}^{**}p < 0.001 = {}^{***}$.

Supplementary Table 1: Chemical structure of tested compounds.

Compound#	Catalog #	Structure/Compound Acronym
PA1	C009	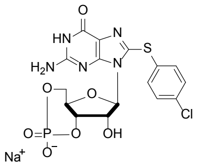 8-pCPT-cGMP
PA2	P003	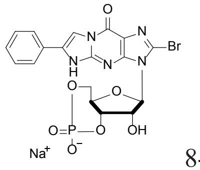 8-Br-PET-cGMP
PA3	B137	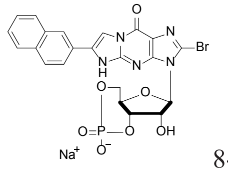 8-Br-(2-N)-ET-cGMP
PA4	G031	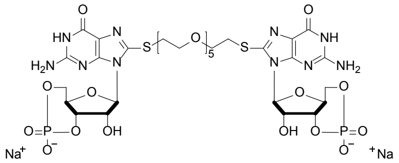 cGMP-8-T-(EO)5-ET-8-cGMP
PA5	G043	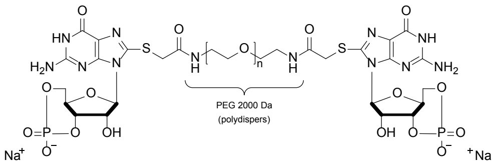 cGMP-8-TMAmd-(PEG pd 2000)-AmdMT-8-cGMP
PA6	B204	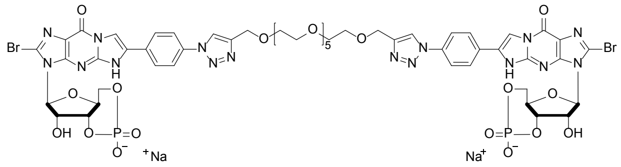 8-Br-cGMP-ETP-p(1-[1,2,3]-Tz-4-)-MeO-(EO)6-Me-p(4-[1,2,3]-Tz-1)-PET-cGMP-8-Br
